# Exploring Cellular Gateways: Unraveling the Secrets of Disordered Proteins within Live Nuclear Pores

**DOI:** 10.21203/rs.3.rs-3504130/v1

**Published:** 2024-01-09

**Authors:** Wenlan Yu, Mark Tingey, Joseph M. Kelich, Yichen Li, Jingjie Yu, Samuel L. Junod, Zecheng Jiang, Ian Hansen, Nacef Good, Weidong Yang

**Affiliations:** Department of Biology, Temple University, Philadelphia, Pennsylvania, USA

**Keywords:** Nuclear pore complexes, Nucleocytoplasmic transport, Super-resolution light microscopy, Intrinsically disordered proteins, Hydrophobic interaction, Live cell imaging, Three-dimensional microscopy imaging, Single-molecule tracking, Nucleoporins, FG-Nups, Nuclear pore structure

## Abstract

Understanding the spatial organization of nucleoporins (Nups) with intrinsically disordered domains within the nuclear pore complex (NPC) is crucial for deciphering eukaryotic nucleocytoplasmic transport. Leveraging high-speed 2D single-molecule tracking and virtual 3D super-resolution microscopy in live HeLa cells, we investigated the spatial distribution of all eleven phenylalanine-glycine (FG)-rich Nups within individual NPCs. Our study reveals a nuanced landscape of FG-Nup conformations and arrangements. Five FG-Nups are steadfastly anchored at the NPC scaffold, collectively shaping a central doughnut-shaped channel, while six others exhibit heightened flexibility, extending towards the cytoplasmic and nucleoplasmic regions. Intriguingly, Nup214 and Nup153 contribute to cap-like structures that dynamically alternate between open and closed states along the nucleocytoplasmic transport axis, impacting the cytoplasmic and nuclear sides, respectively. Furthermore, Nup98, concentrated at the scaffold region, extends throughout the entire NPC while overlapping with other FG-Nups. Together, these eleven FG-Nups compose a versatile, capped trichoid channel spanning approximately 270 nm across the nuclear envelope. This adaptable trichoid channel facilitates a spectrum of pathways for passive diffusion and facilitated nucleocytoplasmic transport. Our comprehensive mapping of FG-Nup organization within live NPCs offers a unifying mechanism accommodating multiple transport pathways, thereby advancing our understanding of cellular transport processes.

## Introduction

The nuclear pore complex (NPC) plays an essential role in eukaryotic cells, acting as the sole gatekeeper to regulate the transport of particles between the nucleus and cytoplasm. The NPC consists of approximately 30 distinct proteins which can be divided into two categories dependent upon the presence of phenylalanine-glycine (FG) repeated amino acid sequence motifs, FG-Nups, and non-FG Nups. Non-FG Nups form the NPC’s primary physical structure, referred to here as the scaffold of the pore. The FG-Nups are anchored to the scaffold of the NPC and form the selectively permeable barrier within the pore^1–4^. FG-Nups commonly exhibit extensive regions of intrinsically disordered structure that encompass multiple FG motifs. Depending upon the distinct amino acid sequence, these FG regions commonly are classified as one of three distinct types: FG, FxFG, or GLFG^2,5,6^. The unique biophysical properties of FG-Nups have been shown to form the selectively permeable barrier within the NPC that allows for the signal-independent passive diffusion of small molecules under about 40–60kDa, while repelling larger particles that are not bound to karyopherins (nuclear transport receptors, NTRs). NTRs function to ferry cargo across the pore by directly interacting with FG-Nups. Current opinion describes this mechanism as being reliant on hydrophobic interactions between the hydrophobic pockets found within the heat-repeat containing structures or similar hydrophobic pockets located on NTRs and the hydrophobic regions on and near the FG motifs within FG-Nups^5,7,8^. FG-Nups have proven to be critical in regulating the essential export processes of mRNAs and pre-ribosomes^9–13^, as well as for the nuclear import of nuclear bound proteins such as transcription factors and other DNA binding proteins^14–30^.

The structural characterization of the NPC’s core scaffold proteins has been extensively investigated using various techniques such as electron microscopy (EM), cryo-electron tomography (cryo-ET), X-ray crystallography, computational modeling, crosslinking mass spectrometry, and super-resolution fluorescence microscopy methods like SIM, STORM, and STED^6,31–42^. Experimental evidence has demonstrated that the structural components of NPC exhibit an octagonal rotational symmetry, resulting in a pore with a transmembrane span of approximately 40 nm and an internal diameter that ranges from approximately 50 nm. Eight cytoplasmic filaments extend approximately 30–90 nm into the cytoplasm, originating from the cytoplasmic sub-region. At the nuclear face of the pore, there is a basket structure that extends around 50–75 nm into the nucleoplasm. The reported total length and molecular weight of the mammalian NPC in the literature are approximately 200 nm and 110 MDa, respectively^1,32,37,43–46^.

In the realm of extensive research on relatively stable scaffold Nups, comprehending the selective permeability barrier formed by dynamic and intrinsically disordered FG-Nups presents a formidable challenge. To shed light on the configuration and role of this vital barrier, various models have emerged, often relying on indirect *in vitro* techniques. One early model proposed a central plug in the NPC based on EM and cryo-electron tomography^47–53^. However, recent studies have not validated this concept, suggesting that observed plugs may be transient, potentially linked to large cargo transit events during EM imaging^3,32,47^. In contrast, alternative models draw insights into FG-Nup organization from protein-protein interactions.

The Gradient model posits an affinity gradient for FG-Nups within the pore, suggesting that NTRs transition from low to high affinity as they traverse the pore^48^. Recent investigations have cast doubt on this gradient, indicating a lack of directionality in FG-Nup interactions with NTRs^8,9,49,50^. The Oily Spaghetti model suggests that hydrophobic interactions among FG-Nups create a spaghetti-like structure, resulting in peripheral clumping and a relatively empty central channel^50–52^. This arrangement allows small molecules to diffuse while necessitating larger molecules to associate with an NTR to overcome the barrier^53^. In contrast, the Selective Phase/Hydrogel model envisions FG-domains forming an organized sieve-like meshwork throughout the central channel^54–55^. This facilitates small molecule diffusion through gaps while obstructing large particles until NTRs dissolve the FG-FG crosslinking, aiding in transport.

The Polymer Brush model, or Virtual Gating model, views the central channel as an entropic barrier for both passive and facilitated transport^56–57^. It relies on electrostatic repulsion to regulate cargo assortment, permitting small molecules to pass while imposing higher entropic costs on larger molecules unless they associate with an NTR^51,53,54^. The Reduction of Dimensionality (ROD) model proposes 2D transport with a dense layer of FG domains lining the NPC’s inner wall^47^. NTRs transport cargo through a 2D random walk along the NPC’s wall, while passive diffusion is confined to a narrow core of the pore. Lastly, the Forest model synthesizes elements from prior models, emphasizing spatial separation between hydrophobic FG-regions and charged regions^42^. It categorizes FG-Nups as “shrubs” or “trees” based on their properties and proposes two transport zones: ‘zone 1’ at the pore center and ‘zone 2’ at the periphery, hinting at multiple pathways for passive diffusion and facilitated transport. In summary, contemporary nuclear transport models primarily diverge in their perspectives on FG Nup arrangement within the native NPC and the mechanisms governing passive diffusion and facilitated transport.

Limited direct evidence on FG organization exists, hindering the ability of researchers to distinguish and advance upon FG organizational models. Currently, a comprehensive representation of the spatial locations for all individual FG-Nups is lacking, and the existing descriptions of FG-Nup locations are based on a combination of various EM and super-resolution studies conducted in different organisms, including Saccharomyces cerevisiae, Xenopus oocytes, Dictyostelium discoideum, and various mammalian cell lines^32,33,45,46,55,56^. EM techniques using fixed or frozen samples have yielded spatial information for several FG-Nups, providing valuable insights into their organization^33, 57–60^. However, FG-Nups display remarkable dynamism, and imaging samples following pre-fixation or freezing captures only one potential conformation of these FG-Nups. This limitation can potentially lead to inaccuracies in assessing their behavior and spatial distribution. Furthermore, the presence of isolated nuclei may hinder the conformational changes of FG-Nups, which are frequently induced by interactions with NTRs^47–49^. Consequently, it is conceivable that the effective range of the NPC may surpass previously reported measurements due to the dynamic and mobile nature of FG-Nups within the *in vivo* pore, in contrast to the relatively static scaffold Nups. Atomic force microscopy (AFM) has provided a dynamic perspective on FG-Nups within the NPC, demonstrating their rapid retraction and elongation on the millisecond scale^57,58^. Moreover, considering that the NPC’s length (~200 nm) is just below the spatial resolution limit of conventional fluorescence microscopy (~250 nm on the x and y plane and ~750 nm in the z dimension), live cell imaging techniques with high spatial and temporal resolution are essential to fully capture the activity and positioning of these disordered and dynamic proteins^55^. In recent research, we have employed fluorescently labeled transport receptors (karyopherins) and yeast FG segments to investigate the mammalian FG selectivity barrier using single-point edge-excitation sub-diffraction (SPEED) microscopy. This approach provided a mapping of distinct transport pathways adopted by importins and exportins and revealed the spatial locations of FG components by measuring FG-FG and FG-NTR interactions within the pore^56^. Although this study helped outline the general locations of FG domains within the nuclear pores of permeabilized cells, the precise spatial locations of individual FG-Nups have not been determined in live cells.

In this manuscript, we undertake a comprehensive examination of the mammalian FG-Nup barrier by combining high-speed super-resolution SPEED microscopy with a HaloTag labeling technique. Our research centers on exploring the spatial positions and dynamic characteristics of individual mammalian FG-Nups within living cell NPCs, shedding light on their three-dimensional (3D) arrangement. Additionally, we map the 3D coordinates of transport pathways taken by various NTRs and passively diffusing molecules through the NPCs in live cells, with the goal of aligning them with the derived 3D organization of FG Nups. Our findings reveal distinct conformations and arrangements of FG-Nups, highlighting the presence of a capped adaptable trichoid channel spanning approximately 270 nm across the nuclear envelope. Within this trichoid channel, we observe unique features of FG-Nups: five FG-Nups are anchored at the scaffold region, forming a central doughnut-shaped channel, while six FG-Nups exhibit greater flexibility and extend outward towards the cytoplasmic or nucleoplasmic regions. Notably, Nup214 and Nup153 contribute to the formation of cap-like structures that alternate between open and closed states along the nucleocytoplasmic transport axis on the cytoplasmic and nuclear sides, respectively. Additionally, Nup98 concentrates at the scaffold region and extends throughout the entire nuclear pore complex, overlapping with other FG-Nups. Furthermore, our findings indicate that these FG-Nups exhibit an organization that aligns well with distinct pathways governing passive diffusion and facilitated nucleocytoplasmic transport in live cells. Building upon the insights derived from our live cell data, we conduct a critical evaluation of existing models by integrating information on FG-Nup organization and single-molecule transport in both live and permeabilized cells. Consequently, we highlight the agreements and discrepancies between the existing models and our novel discoveries. Ultimately, we provide a comprehensive map of FG-Nup organization within live NPCs, offering a more intricate understanding of the nucleocytoplasmic transport mechanism. Our proposed model involves distinct pathways through a capped adaptable trichoid channel (CATCH) within the NPCs, underscoring the dynamic nature of nucleocytoplasmic transport.

## Results

### Labeling FG-Nup Inside the NPCs of Live Mammalian Cells

**Figure 1.**
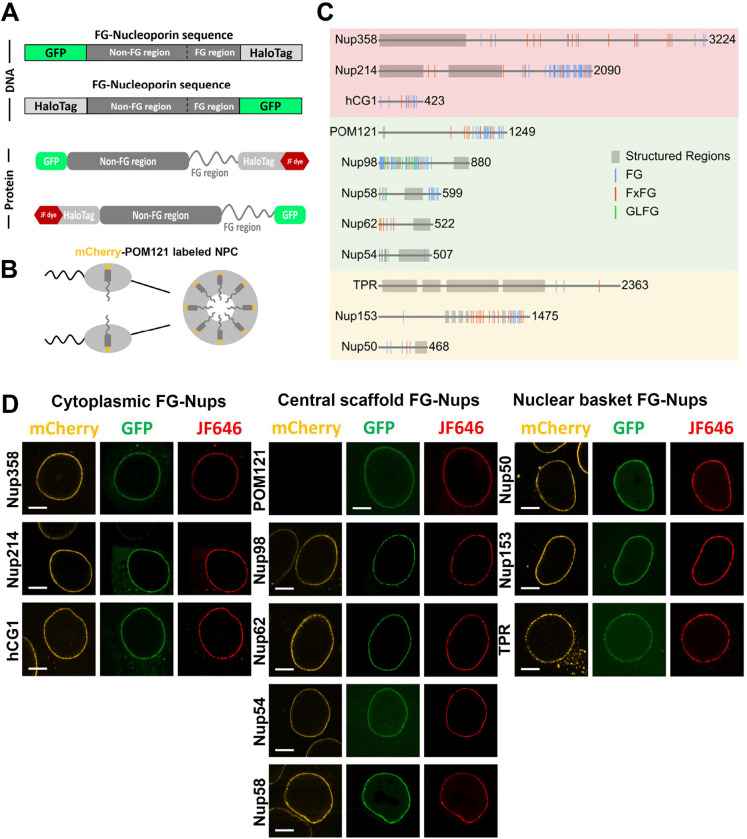
Labeling Strategy and Live Confocal Imaging of FG-Nups Labeled with HaloTag and GFP. **A)** FG Nup constructs illustrating N- and C-terminal labeling strategies. The cartoons delineate the FG-rich and non-FG-rich regions of the proteins and indicate where JF dye binds to the HaloTag. **B)** Schematic depiction of stable expression of mCherry-POM121 in HeLa cells. The rotational symmetrical distribution of POM121 within the NPC allowed us to employ the mCherry signal for NPC centroid determination. **C)** The amino acid sequences of all eleven human FG-Nups, depicting both the FG motifs and more structured regions. FG, FxFG, and GLFP sequences are highlighted in distinct colors. Information about the labeled regions was compiled from the protein structure database and previously published studies^112–122^. **D)** Three-channel live-cell laser scanning confocal image displaying labeled NPCs in live HeLa cells. Bright nuclear envelope (NE) rings are presented in three distinct colors, representing ten FG-Nups. These observations were made by examining NPCs containing either mCherry-POM121 and JF646-HaloTag-FG-Nup-EGFP or mCherry-POM121 and EGFP-FG-Nup-HaloTag-JF646 in live cells. In the case of EGFP-POM121-HaloTag, we observed NE rings in two colors due to the fusion of either EGFP or mCherry at the N terminus. Scale bar: 5 μm.

To enable the visualization and tracking of individual FG-Nups within live NPCs, we employed a labeling strategy combining HaloTag and EGFP. Specifically, we genetically fused HaloTag (~33kDa) to the terminus nearest to the FG domain of each FG-Nup, while EGFP (~27kDa) was attached to the opposite terminus (Fig. 1A–C). Cell-permeable Janelia Fluor^®^ HaloTag^®^ ligands, acting as Halo-specific ligands to recognize the HaloTag located adjacent to FG domains in FG Nups, were introduced into live cells at nanomolar concentrations optimized for single-molecule localization and tracking^59,60^. The presence of GFP served the purpose of confirming successful transfection and assisting in the identification of NPCs that incorporated multiple copies of the Nup of interest before single-molecule tracking (Fig. 1D, Supplementary Fig. S1-S2). Furthermore, to map non-FG-rich termini or FG-rich domains distributed at both ends, we generated constructs with the inverse configuration. In these constructs, we fused HaloTag to the anchoring or more structured segment of the FG-Nup protein, while attaching EGFP to the opposite terminus (Fig. 1A–B). Finally, to accurately determine the centroid position of individual NPCs at the nuclear envelope (NE) of live HeLa cells, we utilized cells that stably expressed mCherry-POM121, a structural Nup fused with mCherry on its N terminus and located at the central scaffold of the NPC (Fig. 1C, Supplementary Fig. S1).

In this study, Janelia Fluor^®^ 646 HaloTag^®^ ligands (JF646) were employed to target the HaloTag, owing to their distinctive design tailored for super-resolution live-cell imaging. Additionally, the far-red spectral properties of JF646 are distinctly separate from those of EGFP and mCherry. Through laser scanning confocal microscopy, we observed bright NE rings in three distinct colors for ten FG-Nups when examining NPCs containing either mCherry-POM121 and JF646-HaloTag-FG-Nup-EGFP or mCherry-POM121 and EGFP-FG-Nup-HaloTag-JF646 in live cells (Fig. 1D). Specifically, in the case of EGFP-POM121-HaloTag, we obtained NE rings in two colors due to the fusion of either GFP or mCherry to the N terminus (Fig. 1D). These observations provide compelling evidence of the successful insertion of labeled FG-Nups into the NPCs and confirm the effective binding of JF646 to the HaloTag-fused FG-Nups. Finally, the viability and overall cellular function of the genetically mutated cells were assessed and compared to wild-type cells through visual inspection of cell morphology under a microscope, as well as monitoring cell proliferation and growth rate. No significant differences were observed, indicating that the mutations did not adversely affect cell health (Fig. 1D).

### Visualizing FG-Nups Anchored within NPCs Using SPEED Microscopy

**Figure 2.**
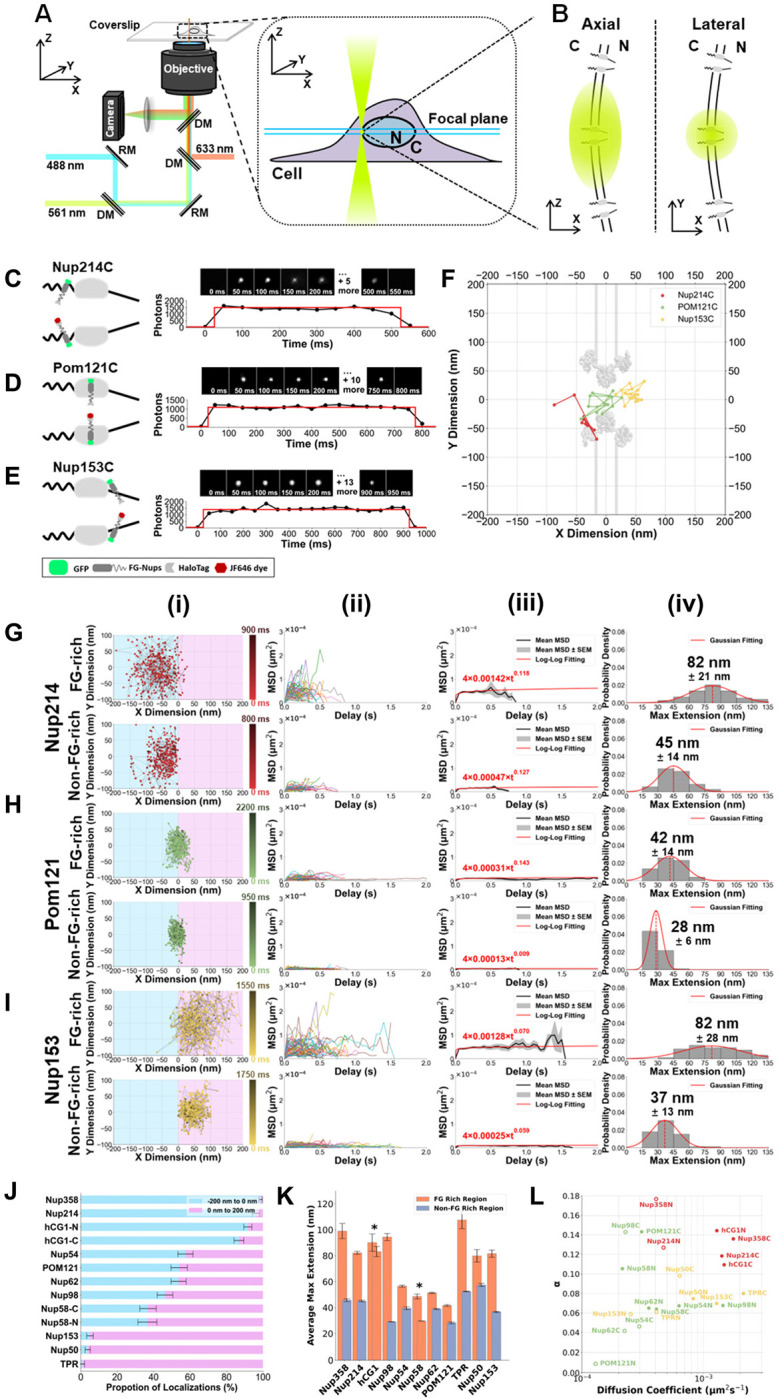
Dynamics of FG Nups within live cell NPCs revealed through 2D single-molecule tracking using SPEED microscopy. **A)** Simplified optical setup for SPEED microscopy. Single-point illumination employs 488-nm, 561-nm, and 633-nm lasers, targeting individual NPCs at the nuclear envelope (NE) equator in live cells. DM: dichroic mirror, RM: reflection mirror, N: Nucleus, C: Cytoplasm. **B)** A single NPC is illuminated at the focal plane of SPEED microscopy, depicted in both axial and lateral dimensions. (N: Nucleus, C: Cytoplasm). **C-E)** Example single-molecule fluorescence are shown for Nup214C located on the cytoplasmic side, POM121C in the central scaffold, and Nup153C in the nuclear basket. The cartoons illustrate the labeling of these FG Nups. The time-series images and their corresponding photobleaching curves indicate the initiation and termination of fluorescence from JF dyes used to label these FG Nups, with the Y-axis representing the photon counts and the X-axis representing time. **F)** Single-molecule tracking and the localization of fluorescent spots in the time-series images, as presented in C-E, generated the 2D single-molecule trajectories for Nup214C (red), POM121C (green), and Nup153C (yellow). These trajectories were then superimposed and overlaid onto the NPC scaffold structure (light grey). The light grey structure represents the NPC scaffold structure obtained through Cryo-EM, sourced from the RCSB Protein Data Bank. It is noteworthy that the structure presented here is the original version publicly available. This structure contains three small fragments of Nup98 and a coiled-coil structure from each of the three central scaffold FG Nups: Nup62, Nup58, and Nup54, in addition to the structural information of scaffold Nups. **G-I)** Mean Squared Displacement (MSD) analyses were conducted on 2D single-molecule trajectories, which were compiled from over fifty single-molecule traces for the FG-rich and non-FG-rich domains of Nup214 (red), POM121C (green), and Nup153C (yellow), respectively. These analyses (ii) of the trajectories (i) provided essential insights, including the extension length (iv), the diffusion coefficient (iii), and the exponent α (iii), as determined by the relationship $MSD(t)=4Dt^{\wedge }\alpha$ for these FG Nups within live cell NPCs. The histogram of extension lengths was fitted with Gaussian functions, facilitating the quantification of the maximum extension length presented as mean ± SD. The blue and pink regions specifically delineate the range from −200 nm to 0 nm (cytoplasmic side) and from 0 nm to 200 nm (nuclear side) along the nucleocytoplasmic transport axis (the X dimension), respectively. In the perpendicular direction (the Y dimension) for both regions, the range extends from −100 nm to 100 nm. **J)** The distribution of localizations for the FG-rich domains of each FG-Nup, evaluated with respect to their 2D spatial locations on the cytoplasmic (blue) or nucleoplasmic (pink) side, is illustrated. The blue and pink areas correspond to the range from −200 nm to 0 nm and from 0 nm to 200 nm along the nucleocytoplasmic transport axis, respectively. **K)** Displays the average maximum extensions (in nm) for each FG-Nup, distinguishing between FG-rich (orange) and non-FG-rich (dark blue) regions. Nups denoted with * contain FG-rich regions (orange) at both ends. **L)** A scatter plot depicts the diffusion of FG Nups positioned on the cytoplasmic side (red), within the central scaffold (green), and on the nuclear side (yellow). The Y-axis represents α, while the X-axis displays the diffusion coefficient (μm^2^/s), presented on a logarithmic scale. N: Nucleus, C: Cytoplasm.

To map HaloTag-fused FG-Nup within the native NPCs, cell-permeable JF646 were introduced to live cells at a final concentration of approximately 0.1–1 nM. Following a 15-minute incubation period, the JF dye molecules penetrated the cells and bound specifically to the HaloTag-fused FG-Nups within the targeted pore. To capture a dynamic picture of individual FG-Nups within live NPCs, high-speed SPEED microscopy was employed. This microscopy technique utilizes point-spread-function (PSF)-limited single-point inclined or vertical illumination patterns in the focal plane, enabling the imaging of a single NPC amidst neighboring NPCs in live cells (Fig. 2A–B, Supplementary Fig. S1). Its unique capabilities allow for the dynamic visualization of molecules within submicrometer biological channels and cavities with rotational symmetry, achieving high spatiotemporal resolutions of ⩽10–20 nm and 0.4–2 ms^11,55,56,61,62^.

Using SPEED microscopy with a detection time of 2 ms per frame and an excitation laser of 632 nm for JF646, we were able to capture and analyze the dynamics of diffusive molecules within live NPCs. Our observations unveiled the presence of three distinct categories of molecules, each characterized by different single-molecule trajectories and residence times within the NPCs. The first group of molecules exhibited rapid movement through the NPCs, completing their passage within approximately 3 ms, corresponding to passive diffusion of free JF dyes through the pore (Table S1, Supplementary Fig. S3). The second group exhibited a more extended residence period, lasting 9–12 ms. These molecules were identified as a subset of unbound JF-labeled Nups that traverse the NPCs (Table S1, Supplementary Fig. S3). The third and final category demonstrated the lengthiest residence time, ranging from 0.2 to 2 seconds before undergoing photobleaching. This group’s characteristics identified them as FG-Nups securely anchored within the NPCs, as depicted in Fig. 2 C–E. The prolonged NPC-dwelling duration of anchored FG-Nups was pivotal in allowing us to effectively filter out the molecules from the first two groups during our data collection process.

In terms of our experimental methodology, a crucial adjustment was made to the frame rate, transitioning from 2 ms per frame to 50 ms per frame. This modification facilitated the precise and selective mapping of anchored FG-Nups without sacrificing the capture of FG Nups’ dynamics within the NPCs of live cells. The rationale behind this change is multifaceted. Firstly, based on the diffusion coefficients exhibited by FG Nups within the native NPC, a frame rate of 50 ms is sufficient for capturing their dynamics (Supplementary Fig. S4-S7). Secondly, the 50-ms detection rate effectively filters out two other transiting groups, ensuring that only anchored FG-Nups within the NPC are collected. Lastly, the chosen frame rate of 50 ms substantially increased photon collection per frame, resulting in an approximate 20-fold rise in photons captured from anchored FG-Nups in the NPCs of live cells. As a result, the precision of single-molecule localization precision improved significantly, achieving a spatial resolution of up to 4 nm, with an average of 7 ± 3 nm across a dataset comprising over 20,000 data points. This high spatial localization precision enabled us to precisely determine the spatial locations of FG Nups in the 2D single-molecule tracking experiments and ensure high reproducibility in 3D configurations of FG-Nup organization. To maintain data integrity, we applied a cutoff localization precision of ≤ 10 nm (Table S2) to filter single-molecule locations, allowing us to further exclude mobile unbound JF-labeled Nups or unbound dye from our experimental results, thereby focusing exclusively on the anchored FG-Nups.

### Distinct dynamic behaviors observed for FG-Nups within live NPCs

In our study, as previously mentioned, we Halo-tagged both the FG-rich terminus and the opposite terminus of each of the eleven FG-Nups in live cells, leading to a total of twenty-two JF dye-HaloTag labeled terminals for these FG Nups. During 2D single-molecule tracking experiments conducted at a frame rate of 50 ms, we recorded between one to two thousand spatial locations for each targeted FG Nup within the NPC. These locations comprised sequences of four to forty continuous frames, collected from approximately fifty trajectories for each labeled terminus of FG Nup. These experiments were conducted across ten different NPCs in ten different live cells (Fig. 2 F–I, Supplementary Fig. S6-S7). Subsequently, we conducted Mean Squared Displacement (MSD) analysis on these single-molecule trajectories (Fig. 2 G–I, Supplementary Fig. S6-S7), which provided us with valuable data, including the extension length, the diffusion coefficient, and the exponent α through the relationship $MSD(t)=4Dt^{\alpha }$. Figure 2 G–I showcases examples of data collected for three distinct sub-NPC locations: Nup153 for the nuclear basket side, POM121 for the scaffold region, and Nup214 for the cytoplasmic side.

First, our findings revealed the dynamic behavior of the FG-rich terminal domains of all eleven FG-Nups, with noticeable variations in their extension lengths (Fig. 2 G–I, Supplementary Fig. S6-S7). It appears that their extension lengths are significantly influenced by their respective positions within the NPC (Fig. 2 G–I, Supplementary Fig. S6-S7). Pooling the average maximum extensions of all FG-Nups (Fig. 2 J–K) unveiled two distinct behaviors: Asymmetric FG-Nups, anchored to the cytoplasmic and nuclear faces of the pore, including Nup358, hCG1, Nup214, Nup153, Nup50 and TPR, displayed greater extension capabilities (ranging from 80–108 nm). In contrast, most symmetric and centrally located FG-Nups – POM121, Nup62, Nup58, and Nup54 - exhibited smaller extension ranges (ranging from 30–57 nm). However, Nup98, an exception among the centrally located FG-Nups, exhibited a significant extension of approximately 94 nm. This observation suggests that its extension is not limited to the central scaffold region, unlike other centrally located FG-Nups. Instead, it appears to traverse nearly the entire nucleocytoplasmic path through the NPC. Notably, hCG1 contains FG motifs throughout its sequence, with both its N and C terminal domains extending to similar lengths of 83 nm and 90 nm, respectively. As expected, Nup358C and TPRC (the FG-rich end of Nup358 and TPR, respectively), showcased the greatest maximum extension at 99 nm and 108 nm, respectively (Supplementary Fig. S6-S7).

In contrast, non-FG-rich terminals had significantly lower extension values compared to FG-rich terminals (Fig. 2K, Table 1), indicating that the presence of FG-domains enables greater flexibility in spatial exploration. Meanwhile, it is worth highlighting that these anchoring or more structural domains also exhibit notable extension ranges, despite their established role in binding to the NPC’s structural proteins. This extension could potentially be linked to conformational alterations within these protein domains or the structural proteins they are anchored to. These observations align with earlier findings, indicating the presence of subtle conformational adjustments, often referred to as “breathing,” within highly structured proteins such as the NPC’s scaffold proteins^63–65^.

Lastly, the MSD analysis revealed that FG-Nups within live cell NPCs exhibit strong subdiffusion as they all have an α below 0.3^66^ (Fig. 2L). Specifically, the relative diffusive relationship of all FG-Nups, including both the FG-rich regions and non-FG-rich regions, was plotted with α on the Y-axis and diffusion coefficient (μm^2^s^−1^) on the X-axis (Fig. 2L). Several interesting observations were made. First, POM121N, the anchoring end lacking FG-rich regions, displayed the least mobility among all non-FG-rich terminals. This discovery is notably intriguing and essential, given that this location serves as the reference point for superimposing the trajectories of all FG-Nups in our measurements. Second, all FG-Nups demonstrated patterns consistent with confined diffusion^67^ (Supplemental Fig. S7). This provides further confirmation that only tethered FG-Nups were imaged and analyzed in this study. It’s noteworthy that TPRC displayed the highest diffusion coefficient but still exhibited an α indicative of confined diffusion. Intriguingly, Nup98N, the FG-rich end of Nup98 actively diffusing beyond the scaffold region along the NPC’s axis, displayed a similar pattern to TPR but on a smaller scale. Lastly, all cytoplasmic Nups displayed an α value greater than 0.1, indicating strong subdiffusion with less confinement (Fig. 2L). The FG-rich ends of these Nups exhibited some of the highest diffusion coefficients, consistent with their long intrinsically disordered regions (IDRs) and the absence of physical constraints from the NPC structure or nuclear membrane. Notably, the length of the IDRs of each FG Nup correlated with its respective diffusion coefficient, with longer FG-rich IDRs associated with higher diffusion coefficients (Fig. 2L). In summary, the above measurements provide compelling evidence for the significant dynamic behavior of the FG domains within FG-Nups, showcasing the remarkable flexibility and adaptability of the FG-Nup architecture within the native NPCs.

### Three-dimensional (3D) spatial distributions of five central FG-Nups within the NPCs in live HeLa cells

**Figure 3.**
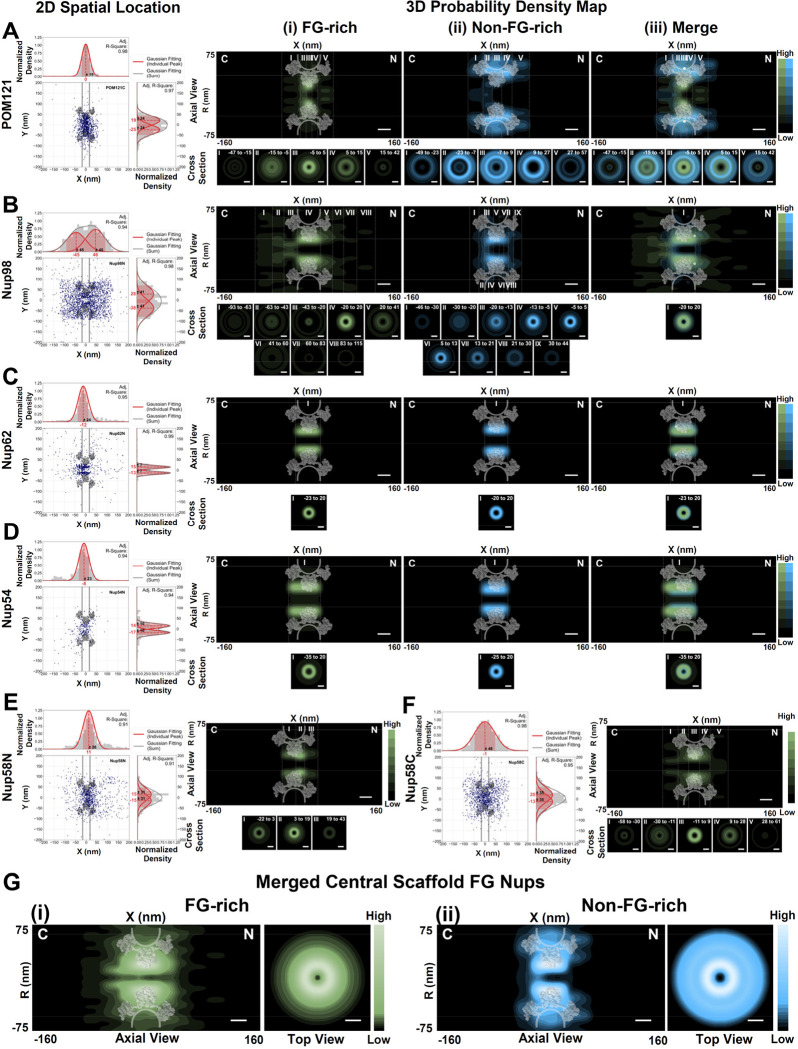
2D Spatial Localizations and 3D Spatial Probability Density Maps for FG Nups Situated at the Central Scaffold of the NPC. **(A-D)** 2D Spatial Localizations of the FG-rich Domains and 3D Spatial Probability Density Maps for both the FG-rich and non-FG-rich domains of POM121, Nup98, Nup62, and Nup54. Normalized 2D spatial density histograms from these 2D spatial localizations were fitted with Gaussian functions along the X and Y dimensions, respectively. The 2D spatial localizations of the non-FG-rich Domains were provided in the Supplementary Information. The 3D Spatial Probability Density Maps are presented in a slice view, highlighting dimensions along the axial (X dimension, representing the cytoplasmic transport axis) and cross-sectional (R dimension, perpendicular to the cytoplasmic transport axis) orientations. These maps are provided for the FG-rich domains (in green), the non-FG-rich domains (in blue), and the composite map of both FG-rich and non-FG-rich domains (in blue-green) for these FG Nups. The light grey structure depicts the NPC scaffold, including small fragments of the central scaffold FG Nups (Nup98, Nup62, Nup58, and Nup54), obtained through Cryo-EM and sourced from the RCSB Protein Data Bank. Roman numerals indicate cross-section slices representing probability density along the R dimension. The color bar, ranging from dark to bright, represents the density change from low to high. Scale bar: 20 nm. N: nucleus. C: Cytoplasm. **(E-F)** 2D Spatial Localizations and 3D Spatial Probability Density Maps for Nup85C and Nup58N. Normalized 2D spatial density histograms from these 2D spatial localizations were fitted with Gaussian functions along the X and Y dimensions, respectively. The slice views of 3D spatial probability density maps are presented in axial (X dimension, representing the cytoplasmic transport axis) and cross-sectional (R dimension, perpendicular to the cytoplasmic transport axis) views. The light grey structure depicts the NPC scaffold, including small fragments of the central scaffold FG Nups (Nup98, Nup62, Nup58, and Nup54), obtained through Cryo-EM and sourced from the RCSB Protein Data Bank. Roman numerals indicate cross-section slices that illustrate probability density along the R dimension. The color bar, ranging from dark to bright, represents the density change from low to high. Scale bar: 20 nm. N: nucleus. C: Cytoplasm. **(G)** Overlap of the FG-rich and non-FG-rich domains of the five FG Nups located at the central scaffold of the NPC, shown in both the axial and top views. The light grey structure depicts the NPC scaffold, including small fragments of the central scaffold FG Nups (Nup98, Nup62, Nup58, and Nup54), obtained through Cryo-EM and sourced from the RCSB Protein Data Bank. The color bar, ranging from dark to bright, represents the density change from low to high. Scale bar: 20 nm. N: nucleus. C: Cytoplasm.

**Figure 4.**
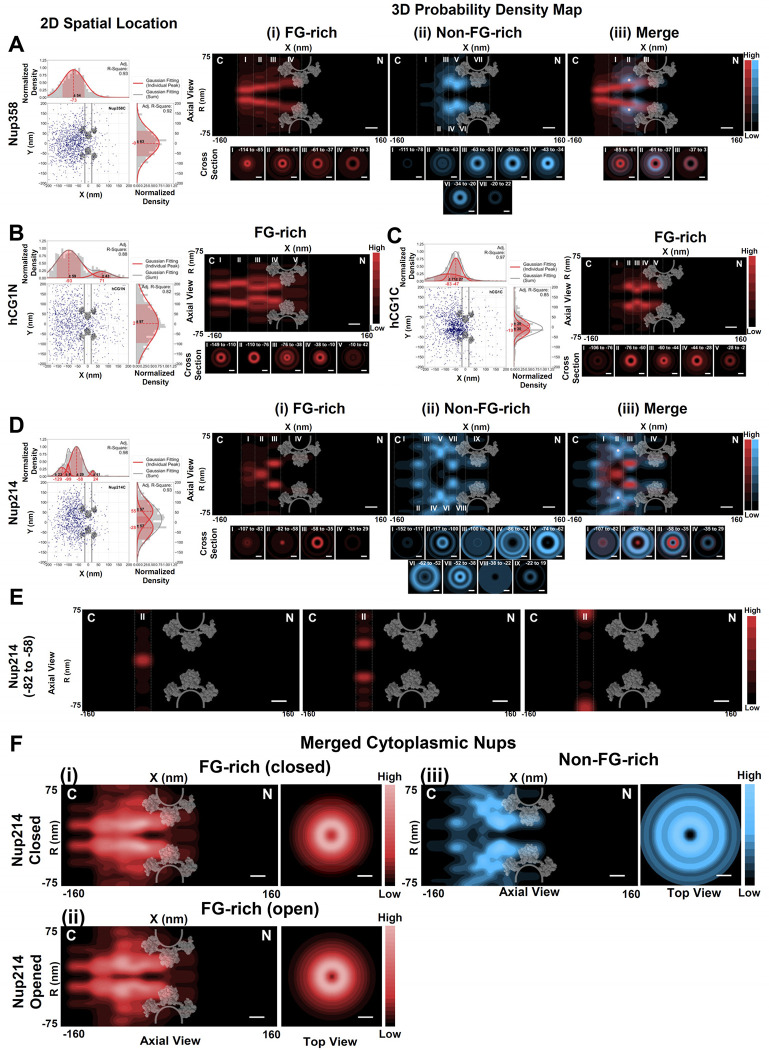
2D Spatial Localizations and 3D Spatial Probability Density Maps for FG Nups Situated on the Cytoplasmic Side of the NPC. **(A)** 2D Spatial Localizations of the FG-rich Domains and 3D spatial probability density maps for both the FG-rich and non-FG-rich domains of Nup358. Normalized 2D spatial density histograms from the 2D spatial localizations were fitted with Gaussian functions along the X and Y dimensions, respectively. The 2D spatial localizations of the non-FG-rich Domains were provided in the Supplementary Information. The slice views of 3D spatial probability density saps are presented in axial (X dimension, representing the cytoplasmic transport axis) and cross-sectional (R dimension, perpendicular to the cytoplasmic transport axis) views for (i) the FG-rich domains (red), (ii) the non-FG-rich domains (blue), and (iii) the combined map of the FG-rich and non-FG-rich domains (red-blue) for Nup358. The light grey structure depicts the NPC scaffold, including small fragments of the central scaffold FG Nups (Nup98, Nup62, Nup58, and Nup54), obtained through Cryo-EM and sourced from the RCSB Protein Data Bank. Roman numerals indicate cross-section slices representing probability density along the R dimension. The color bar, ranging from dark to bright, represents the density change from low to high. Scale bar: 20 nm. N: nucleus. C: Cytoplasm. **(B-C)** 2D Spatial Localizations and 3D spatial probability density maps for hCG1C and hCG1N. Normalized 2D spatial density histograms from these 2D spatial localizations were fitted with Gaussian functions along the X and Y dimensions, respectively. The slice views of 3D spatial probability density maps are presented in axial (X dimension, representing the cytoplasmic transport axis) and cross-sectional (R dimension, perpendicular to the cytoplasmic transport axis) views. The light grey structure depicts the NPC scaffold, including small fragments of the central scaffold FG Nups (Nup98, Nup62, Nup58, and Nup54), obtained through Cryo-EM and sourced from the RCSB Protein Data Bank. Roman numerals indicate cross-section slices that illustrate probability density along the R dimension. The color bar, ranging from dark to bright, represents the density change from low to high. Scale bar: 20 nm. N: nucleus. C: Cytoplasm. (**D**) 2D Spatial Localizations of the FG-rich Domains and 3D spatial probability density maps for both the FG-rich and non-FG-rich domains of Nup214. Normalized 2D spatial density histograms from the 2D spatial localizations were fitted with Gaussian functions along the X and Y dimensions, respectively. The 2D spatial localizations of the non-FG-rich Domains were provided in the Supplementary Information. The slice views of 3D spatial probability density saps are presented in axial (X dimension, representing the cytoplasmic transport axis) and cross-sectional (R dimension, perpendicular to the cytoplasmic transport axis) views for (i) the FG-rich domains (red), (ii) the non-FG-rich domains (blue), and (iii) the combined map of the FG-rich and non-FG-rich domains (red-blue) for Nup214. The light grey structure depicts the NPC scaffold, including small fragments of the central scaffold FG Nups (Nup98, Nup62, Nup58, and Nup54), obtained through Cryo-EM and sourced from the RCSB Protein Data Bank. Roman numerals indicate cross-section slices representing probability density along the R dimension. The color bar, ranging from dark to bright, represents the density change from low to high. Scale bar: 20 nm. N: nucleus. C: Cytoplasm. (**E**)The plug-like structure formed by Nup214C undergoes transitions between an engaged (closed) state and two disengaged (open) states, with varying probabilities and dimensions in diameter. The color bar, ranging from dark to bright, represents the density change from low to high. Scale bar: 20 nm. N: nucleus. C: Cytoplasm. **(F)** Overlap of the FG-rich and non-FG-rich domains of the three FG Nups situated on the cytoplasmic side of the NPC, presented in both the axial and top views. The open and closed states of Nup214C were separately incorporated into the maps of FG-rich domains (i and iii). The light grey structure depicts the NPC scaffold, including small fragments of the central scaffold FG Nups (Nup98, Nup62, Nup58, and Nup54), obtained through Cryo-EM and sourced from the RCSB Protein Data Bank. The color bar, transitioning from dark to bright, signifies changes in density from low to high. Scale bar: 20 nm. N: nucleus. C: Cytoplasm.

**Figure 5.**
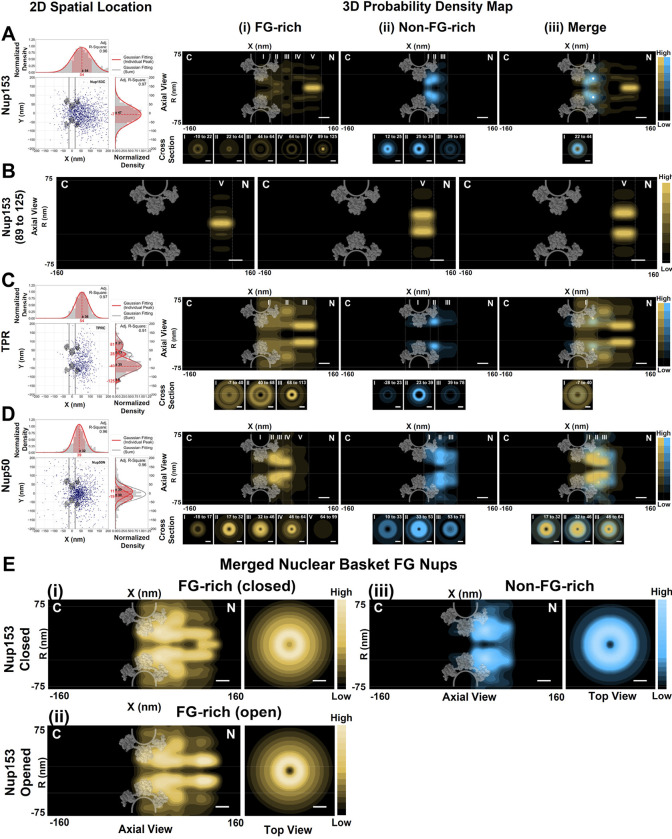
2D Spatial Localizations and 3D Spatial Probability Density Maps for FG Nups Situated on the Nuclear Side of the NPC. **(A)** 2D Spatial Localizations of the FG-rich Domains and 3D Spatial Probability Density Maps for both the FG-rich and non-FG-rich domains of Nup153. Normalized 2D spatial density histograms from the 2D spatial localizations were fitted with Gaussian functions along the X and Y dimensions, respectively. The 2D spatial localizations of the non-FG-rich Domains were provided in the Supplementary Information. The slice views of 3D spatial probability density maps are presented in axial (X dimension, representing the cytoplasmic transport axis) and cross-sectional (R dimension, perpendicular to the cytoplasmic transport axis) views for the FG-rich domains (yellow), the non-FG-rich domains (blue), and the combined map of the FG-rich and non-FG-rich domains (yellow-blue) for these FG Nups. The light grey structure depicts the NPC scaffold, including small fragments of the central scaffold FG Nups (Nup98, Nup62, Nup58, and Nup54), obtained through Cryo-EM and sourced from the RCSB Protein Data Bank. Roman numerals indicate cross-section slices representing probability density along the R dimension. The color bar, ranging from dark to bright, represents the density change from low to high. Scale bar: 20 nm. N: nucleus. C: Cytoplasm. **(B)** The plug-like structure formed by Nup153C undergoes transitions between an engaged (closed) state and two disengaged (open) states, with varying probabilities and dimensions in diameter. The light grey structure depicts the NPC scaffold, including small fragments of the central scaffold FG Nups (Nup98, Nup62, Nup58, and Nup54), obtained through Cryo-EM and sourced from the RCSB Protein Data Bank. Roman numerals indicate cross-section slices representing probability density along the R dimension. The color bar, ranging from dark to bright, represents the density change from low to high. Scale bar: 20 nm. N: nucleus. C: Cytoplasm. **(C-D)** 2D Spatial Localizations of the FG-rich Domains and 3D Spatial Probability Density Maps for both the FG-rich and non-FG-rich domains of TPR and Nup50. Normalized 2D spatial density histograms from these 2D spatial localizations were fitted with Gaussian functions along the X and Y dimensions, respectively. The 2D spatial localizations of the non-FG-rich Domains were provided in the Supplementary Information. The slice views of 3D spatial probability density maps are presented in axial (X dimension, representing the cytoplasmic transport axis) and cross-sectional (R dimension, perpendicular to the cytoplasmic transport axis) views for the FG-rich domains (yellow), the non-FG-rich domains (blue), and the combined map of the FG-rich and non-FG-rich domains (yellow-blue) for these FG Nups. The light grey structure depicts the NPC scaffold, including small fragments of the central scaffold FG Nups (Nup98, Nup62, Nup58, and Nup54), obtained through Cryo-EM and sourced from the RCSB Protein Data Bank. Roman numerals indicate cross-section slices representing probability density along the R dimension. The color bar, ranging from dark to bright, represents the density change from low to high. Scale bar: 20 nm. N: nucleus. C: Cytoplasm. **(E)** Overlap of the FG-rich and non-FG-rich domains of the three FG Nups situated on the nuclear side of the NPC, presented in both the axial and top views. The open and closed states of Nup153C were separately incorporated into the maps of FG-rich domains (i and ii). The light grey structure depicts the NPC scaffold, including small fragments of the central scaffold FG Nups (Nup98, Nup62, Nup58, and Nup54), obtained through Cryo-EM and sourced from the RCSB Protein Data Bank. The color bar, transitioning from dark to bright, signifies changes in density from low to high. Scale bar: 20 nm. N: nucleus. C: Cytoplasm.

Beyond the observed dynamics, the trajectories of FG-Nups within native NPCs also provide 2D super-resolution spatial data, revealing distinct distributions of FG-rich and non-FG-rich termini for each FG-Nup (Fig. 3–5, Supplementary Fig. S8). Specifically, the centrally anchored FG-Nups (POM121, Nup98, Nup62, Nup54, and Nup58) exhibit relatively symmetric distributions between the cytoplasmic and nucleoplasmic halves of the NPC along its axial and radial dimensions (Fig. 3 A–F). On the other hand, asymmetric FG-Nups, concentrated their spatial locations either on the cytoplasmic or nuclear sides of the NPC, generally do not extend to the opposing side of the pore (Fig. 4 A–D and Fig. 5 A–C). Notably, hCG1N exhibits approximately 10% of its locations on the nuclear face of the NPC (Fig. 4 B–C), making it the only asymmetric FG-Nup capable of spanning across the pore.

The above 2D localizations provide valuable insights into the relative arrangement within the NPC concerning the localization of FG-rich and non-FG-rich termini of FG Nups. However, it is crucial to acknowledge that 2D data represents a projection of 3D information, which can potentially lead to misinterpretations (Supplementary Figure S9-S10)^68–70,121^. Importantly, several existing models of NPC organization propose that the entire NPC’s channel may be filled with FG-domains of FG-Nups (uniform, central and biomodel configurations in Supplementary Fig. S10B) ^68–70,121^. Relying solely on 2D data could inadvertently support this notion (Y-dimension histogram of 2D data in Supplementary Fig. S10B). As demonstrated later, this assumption will be refuted when we reveal their 3D structures (Fig. 3–5, R-dimension histogram of 3D structure in Supplementary Fig. S10)^61,62,68–70,121^.

To surmount this limitation and achieve a more profound comprehension of the NPC organization, we have developed a 2D to 3D transformation algorithm. This advanced algorithm enables us to infer the most probable locations of macromolecules within the NPC in three dimensions, as elucidated in Supplementary Figure S9-S11^55,56,61,62,70,121^. By capitalizing on the NPC’s inherent cylindrical symmetry, the 2D to 3D transformation algorithm converts 2D projected data into a 3D probability density map, as portrayed in Supplementary Figure S14-S20. This transformative approach has enabled us to construct a virtual 3D super-resolution microscopy system, facilitating the mapping of 3D nucleocytoplasmic transport pathways for a wide array of cargos. These include passive diffusion of small molecules as well as the facilitated transport of proteins and mRNAs, as described in previous studies^10,55,56,61,62,70,71^.

To surmount this limitation and achieve a more profound comprehension of the NPC organization, we have developed a 2D to 3D transformation algorithm. By capitalizing on the NPC’s inherent cylindrical symmetry, the post-localization 2D to 3D transformation algorithm converts 2D projected data into a 3D probability density map, as portrayed in Supplementary Figure S9–11. This transformation process has been rigorously validated in various systems with inherent rotational symmetry, such as glass nanocapillary tubes, primary cilia, microtubules, and NPCs^55,56,61,62,70,121^. Notably, the accuracy and reproducibility of 3D probability density maps generated through this transformation in the NPC have been extensively validated under diverse experimental conditions and Monte Carlo simulations^55,56,61,62,70,121^. We meticulously examined several critical factors affecting the transformation process, including single-molecule localization precision, the number of 2D spatial locations, imperfect radial symmetry of the NPC, NPC orientation or rotation at the nuclear envelope, and the labeling efficiency of targeted substrates within the NPC. Detailed in Supplementary Figures S14-S20, optimized parameter quantification ensures the reliable generation of 3D probability density maps for both anchored proteins within the NPC and moving molecules through the NPCs. By employing these optimized parameters, this transformative approach has empowered us to construct a virtual 3D super-resolution microscopy system. This system facilitates the mapping of 3D nucleocytoplasmic transport pathways for a diverse range of cargos, including the passive diffusion of small molecules, as well as the facilitated transport of proteins and mRNAs, as previously described in studies^10,55,56,61,62,70,71^.

Through the utilization of the 2D to 3D transformation algorithm, we initially generated 3D spatial probability density maps for five FG-Nups located in the central scaffold of the NPC, specifically POM121, Nup98, Nup62, Nup54, and Nup58 (Supplementary Movies S1-S7). Consistent with dynamic measurements of these FG Nups, the 3D spatial distribution maps suggest that their FG-rich termini typically occupy larger spatial volumes compared to their non-FG-rich ends (Fig. 3). Notably, the 3D spatial distributions unveiled intriguing insights regarding their FG domains. First, the FG domains of these central scaffold FG Nups exhibit their highest spatial density within the NPC’s scaffold. Each of these five FG Nups consistently displays a doughnut-shaped structure, with both their FG-rich domains and non-FG-rich domains forming this structure in proximity to the scaffold (Fig. 3A–F). This structural feature spans from approximately −20nm to 20nm, corresponding to the thickness of the double-membrane nuclear envelope. FG-rich domains shape a less-occupied axial channel with a diameter of approximately 7 nm (Fig. 3G, Supplementary Movies S7)^71–75^. Second, these FG Nups can extend in varying lengths into both the cytoplasmic and nuclear sides of the NPC. Specifically, Nup62 and Nup54 predominantly remain within the scaffold region with minimal extensions (Fig. 3 C–D, Supplementary Movies S3-S4). In contrast, the FG domains of Nup58 exhibit substantial overlap with these FG Nups in the central scaffold but extend approximately 40 nm into both directions (Fig. 3 E–F, Supplementary Movies S5-S6). Intriguingly, POM121, serving as both a transmembrane protein and an FG Nup, also extends its FG domains approximately 40 nm into both ends, while forming a doughnut-shaped structure with Nup98, Nup62, Nup54, and Nup58 at the scaffold (Fig. 3A, Supplementary Movies S1, S7). Most remarkably, the FG domains of Nup98 possess the longest extension length, extending about 70 nm into the cytoplasmic side and 95 nm into the nuclear side of the NPC (Fig. 3B, Supplementary Movies S2). Such extensive extensions suggest that Nup98’s FG domains likely overlap with those of all other FG domains across the NPC.

To delve deeper into this possibility, we examined the overlap between all FG-Nup terminal domains (N and C) using both Pearson’s correlation coefficient and Spearman’s rank correlation coefficient (Supplementary Table S3-S7). As expected, the overlap predominantly involves Nups localized within the same region, resulting in positive correlations primarily among Nups within the same group. However, Nup98 stands out as an exception, displaying overlaps with other FG Nups in both the cytoplasmic and nucleoplasmic regions (Supplementary Table S3-S7). Notably, GLFG-containing Nup98 demonstrates notably stronger positive correlations with the central and nuclear basket FG Nups compared to those on the cytoplasmic side, further highlighting that the exploration of Nup98 extends into the nuclear basket with some regularity. Previous studies have reported that GLFG-containing Nup98 displays strong positive correlations with all three nuclear basket Nups: Nup50, Nup153, and TPR ^35,76,77^. Furthermore, when evaluating the correlation between Nup98 and the nuclear basket Nups in all three dimensions (XY and R dimension), the correlation calculations reveal a positive correlation among all three nuclear basket Nups (Supplementary Table S6), while a positive relationship exists only between Nup98 and both Nup153 and Nup50, as determined by Pearson’s correlation coefficient (Supplementary Table S7).

Finally, the combined FG-rich and non-FG-rich domains of these central scaffold FG Nups collectively adopt a doughnut-shaped structure, with FG domains concentrated at the peripheral region encompassing an almost vacant central channel with a diameter of approximately 7 nm (Fig. 3G, Supplementary Movie S7). These findings align with previous simulations and measurements^44,55,78–83^ and lend support to specific existing models of FG-Nup organization within the NPC, as elaborated in the subsequent discussion section.

### 3D spatial distributions of the FG-Nups on the cytoplasmic and nuclear sides of the NPCs in live HeLa cells

Next, we further expanded our measurements to encompass the FG Nups situated on the cytoplasmic side of the NPC, specifically Nup358, Nup214, and hCG1. Our 3D probability density maps reveal distinct distributions within these FG Nups, distinguishing between their FG-rich and non-FG-rich domains. Notably, the FG-rich domains extend more deeply toward the cytoplasm, while the non-FG-rich regions remain closer to the central scaffold (Fig. 4). For Nup358, the largest FG Nup on the cytoplasmic side, its FG domains form a V-shaped tube-like structure, with a smaller opening extending approximately 110 nm into the cytoplasm (Fig. 4A, Supplementary Movies S8). In the case of hCG1, which contains FG repeats spanning from its N- to C-terminus, we mapped the 3D distributions for both hCG1N and hCG1C. Intriguingly, their FG domains extend even further into the cytoplasm, with distances of approximately 150 nm and 106 nm, respectively, forming tube-like structures (Fig. 4 B–C, Supplementary Movies S9-S10). Notably, Nup214 exhibits a more complex structural arrangement (Fig. 4D, Supplementary Movies S11). Firstly, the non-FG-rich end of Nup214, referred to as Nup214N, occupies a larger spatial volume than its FG-rich end, Nup214C, extending approximately 150 nm into the cytoplasm, compared to about 100 nm for Nup214C. Secondly, unlike the prevailing peripheral positions of Nup358, hCG1, and the five FG Nups within the central scaffold of the NPC, the FG-rich domains of Nup214 exhibit a significant spatial concentration at the central cytoplasmic end along the nucleocytoplasmic transport axis (Fig. 4 D–E). Furthermore, we observed dynamic fluctuations in the plug-like structure formed by Nup214C, which undergoes transitions between an engaged (closed) state, occurring with a probability of approximately 42%, and a disengaged (open) state, with a likelihood of around 58%. The open state further comprises two distinct opening sizes, with radii measuring approximately 29 nm and 67 nm, respectively (Fig. 4E). Finally, the combined mapping of these three cytoplasmic-side FG Nups collectively gives rise to a cap-like structure on the cytoplasmic side of the NPC (Fig. 4F, Supplementary Movies S12). This structure can transition between an open and closed state, primarily driven by conformational changes in Nup214C (Fig. 4F i-ii). Our prior research, as well as other investigations, has proposed that competition among NTRs for interactions with FG Nups can trigger significant conformational alterations in FG Nups^56,84–86^.

This unique cap-like structure is not exclusive to Nup214C when we expanded our measurements to include the FG Nups located on the nuclear side of the NPC. As depicted in Figure 5A, the FG domains of Nup153 (Nup153C) also exhibit a similar cap-like structure (Supplementary Movies S13). In detail, along the nucleocytoplasmic transport axis extending into the nucleus, Nup153C contributes a significant spatial density, predominantly concentrated at the right-center of the axis. The densely packed FG-rich domains of Nup153C also undergo transitions between open and closed states (Fig. 5B). However, in contrast to Nup124C, the probability of being in the open state for Nup153C is approximately 40%, with a 60% likelihood of remaining in the closed state. Additionally, its open state consists of two distinct radii, measuring approximately 10 nm and 20 nm (Fig. 5B). Conversely, the other two nuclear-side FG Nups, namely Nup50 and TPR, do not display such a distinctive structure or dynamic changes (Fig. 5C–D, Supplementary Movies S14-S15). Instead, they adopt a tube-like structure, consistent with the observed structure of most FG Nups within live NPCs. Once again, for all these nuclear basket FG Nups, it is notable that their FG-rich domains extend deeper into the nucleus, while their non-FG-rich regions remain closer to the central scaffold (Fig. 5 (ii)-(iii)). Remarkably, Nup153, TPR, and Nup50 exhibit varying degrees of extension into the nucleus, with distances ranging from approximately 100 to 125 nm. What is particularly intriguing is that despite their distinct 3D presentations, when combined, these FG Nups collectively form a shape reminiscent of a nuclear basket (Fig. 5E, Supplementary Movies S16), bearing similarities to the structure mapped by electron microscopy^44,87–90^.

### The spatial organization of FG Nups corresponds to the distinct pathways taken by passive diffusion and facilitated transport through the NPC

**Figure 6.**
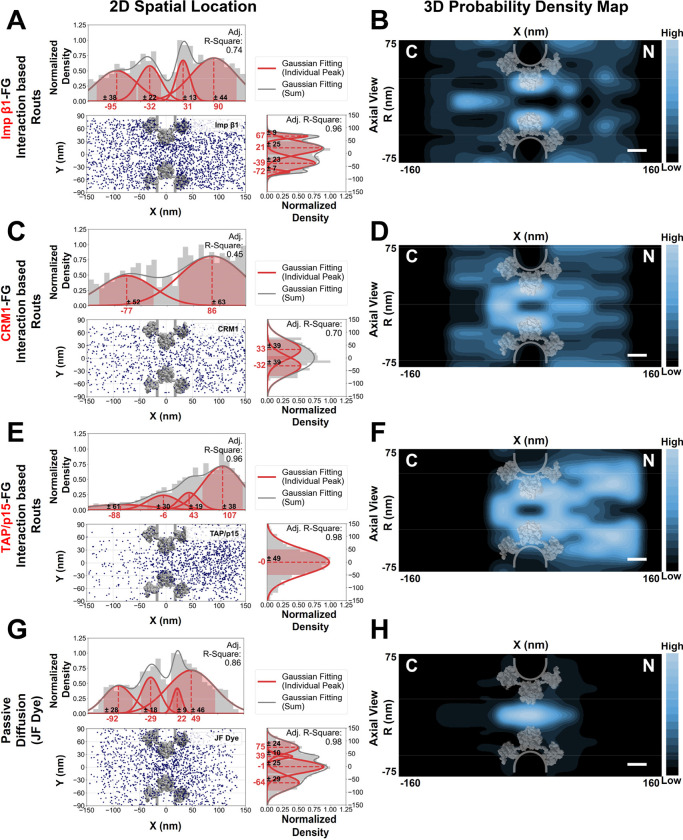
3D Transport Pathways Utilized by Different NTRs and Transiting Macromolecules through the NPCs in Live Cells. (A) 2D spatial locations of EGFP-labeled Importin β1 transport through the NPCs in live cells. Normalized 2D spatial density histograms from the 2D spatial localizations were fitted with Gaussian functions along the X and Y dimensions, respectively. B) 3D Transport Route of Importin β1 through the NPC. The slice view of 3D spatial probability density map of Importin β1 (blue) was oberlaid with the NPC scaffold structure (light grey). The light grey structure depicts the NPC scaffold, including small fragments of the central scaffold FG Nups (Nup98, Nup62, Nup58, and Nup54), obtained through Cryo-EM and sourced from the RCSB Protein Data Bank. (C) 2D spatial locations of EGFP-labeled CRM1 transport through the NPCs in live cells. Normalized 2D spatial density histograms from the 2D spatial localizations were fitted with Gaussian functions along the X and Y dimensions, respectively. (D) 3D Transport Route of CRM1 through the NPC. The slice view of 3D spatial probability density map of CRM1 (blue) was oberlaid with the NPC scaffold structure (light grey). (E) 2D spatial locations of EGFP-labeled TAP/p15 export through the NPCs in live cells. Normalized 2D spatial density histograms from the 2D spatial localizations were fitted with Gaussian functions along the X and Y dimensions, respectively. (F) 3D Export Route of TAP/p15 through the NPC. The slice view of 3D spatial probability density map of Importin β1 (blue) was oberlaid with the NPC scaffold structure (light grey). (G) 2D spatial locations of JF dyes diffuse through the NPCs in live cells. Normalized 2D spatial density histograms from the 2D spatial localizations were fitted with Gaussian functions along the X and Y dimensions, respectively. (H) 3D Passive Diffusion Path of JF Dyes through the NPC. The slice view of 3D spatial probability density map of JF dyes (blue) was oberlaid with the NPC scaffold structure (light grey). Scale bar: 20 nm. N: nucleus. C: cytoplasm.

The preceding measurements offer valuable insights into the distinctive dynamics and spatial arrangements of the eleven FG Nups within the native NPCs in living cells. It is widely recognized that these FG Nups collectively functions as a pivotal selectivity barrier, facilitating the transport of proteins and RNAs in response to signals, as well as the passive diffusion of small molecules through the NPC, irrespective of signals. To delve deeper into the interplay between pathways governed by passive diffusion, facilitated transport through NPCs, and the observed FG-domain paved routes, we charted the 3D transport pathways for small molecules and several primary NTRs, including Importin β1 (Imp β1), CRM1, and TAP/p15, within live cells (Fig. 6). This was accomplished using methodologies akin to those employed for studying FG Nups. Specifically, these NTRs were tagged with EGFP at their C termini, positioned away from their known binding sites on the surfaces of these receptors.

**Figure 7.**
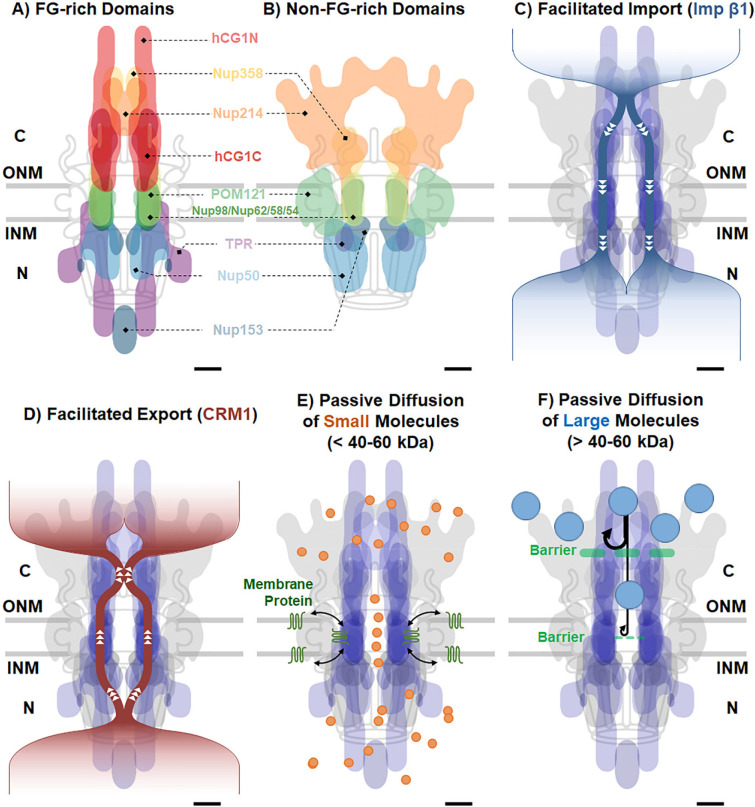
Distinct Pathways through the Capped Adaptable Trichoid Channels (CATCH) within the NPC (A) An illustration depicting the native configuration of FG-rich domains of all eleven FG Nups in the NPC of live cells. N: nucleus. C: Cytoplasm. ONM: Outer Nuclear Membrane. INM: Inner Nuclear Membrane. (B) An illustration depicting the native configuration of non-FG-rich domains of all eleven FG Nups in the NPC of live cells. (C) The facilitated import pathway utilized by Importin-β1 is closely associated with the configuration of FG-rich domains in all eleven FG Nups. Arrows illustrate the import process rather than implying unidirectional movement. N: nucleus. C: Cytoplasm. ONM: Outer Nuclear Membrane. INM: Inner Nuclear Membrane. (D) The facilitated export pathway employed by CRM1 is intricately linked to the configuration of FG-rich domains in all eleven FG Nups. Arrows depict the export process, avoiding the implication of unidirectional movement. N: nucleus. C: Cytoplasm. ONM: Outer Nuclear Membrane. INM: Inner Nuclear Membrane. (E) An illustration depicting the passive diffusion of small molecules, both soluble and membrane-bound (< 40–60 kDa), through the configuration of FG-rich domains of all eleven FG Nups. (F) An illustration depicting the passive diffusion of large molecules (> 60 kDa) encountering barriers (green lines) inhibiting their passive diffusion. Scale bar: 20 nm. N: nucleus. C: Cytoplasm. ONM: Outer Nuclear Membrane. INM: Inner Nuclear Membrane.

We firstly mapped the 3D transport routes for Imp β1 in living cells (Fig. 6 A–B, Fig. 7C, Supplementary Movies S17). Imp β1 plays a pivotal role as a nuclear transport receptor, facilitating the translocation of molecules bearing Nuclear Localization Signals (NLS) from the cytoplasm to the nucleus. It actively engages with nearly all eleven FG Nups^4^. Notably, we observed a significant concentration of Imp β1 molecules along the nucleocytoplasmic transport axis on the NPC’s cytoplasmic side, particularly overlapping with the “close” state of the central ‘plug’ contributed by Nup214C. Subsequently, Imp β1 molecules navigated through the FG-rich periphery surrounding the sparsely occupied central axial channel, primarily composed of FG domains of the five central scaffold FG Nups (Fig. 6 A–B). On the nuclear side of the NPC, Imp β1 positions closely aligned with the nuclear basket structure formed by Nup153, Nup50, and TPR. However, unlike their behavior on the cytoplasmic side, the spatial distrubution of Imp β1 molecules align well with the “open” state of the ‘plug’ structure contributed by Nup153 (Fig. 5 E(ii), Fig. 6B).

To provide a comprehensive depiction of the FG barrier within the NPC, we integrated 3D maps encompassing both the FG-rich and non-FG-rich domains of each FG Nup, resulting in a holistic representation (Fig. 7 A–B). Altogether the eleven FG Nups form a capped, adaptable trichoid channel spanning approximately 270 nm in total length and extending to about 100 nm in diameter at both ends. As depicted in Fig. 7C, the transport route followed by Imp β1 through the NPC in live cells appears to align closely with the spatial arrangement of FG Nups.

Subsequently, our live-cell mapping of the 3D export routes for two pivotal nuclear export receptors, CRM1 and TAP-p15, which facilitate the export of messenger RNA (mRNA) and, in the case of CRM1, also proteins from the nucleus through the NPC, reveals that their export pathways closely align with the FG-rich periphery of the central scaffold (Fig. 6 C–F, Fig. 7D, Supplementary Movies S18-S19). Remarkably, both CRM1 and TAP-p15 exhibit spatial distributions on both ends that seem to overlap with the ‘close’ state of these cap-like structures (Fig. 6 C–D). However, their pathways are not identical, particularly when they interact on both ends of the NPC (Fig. 6 E–F, Fig. 7D). This observation of similarities and differences in their pathways may support their distinct roles as the two primary exportins responsible for aiding different sets of mRNAs in exiting the nucleus^8,10,91–94^.

Finally, the 3D paths for passive diffusion of small and large particles are in line with the organization of FG Nups as well (Fig. 6 G–H, Fig. 7 E–F, Supplementary Fig. S12-S13, Supplementary Movies S20-S21). Observations in both live and permeabilized cells sugegst that small molecules (< 40–60kDa) traverse through the cap-like structure and proceed through the less-FG-occupied central axial channel with a dimater of about 7nm, while larger particles (> 60kDa) initially encounter obstruction within the cap-like structure at the cytoplasmic side and subsequently face hindrance within the narrowest central channel. It is noteworthy that membrane proteins transport their transmembrane domains from the outer membrane of the NE to the inner membrane through a mechanism referred to as the lateral retention-diffusion model^95,96^. These proteins utilize peripheral channels formed between the scaffold Nups of the NPC and the NE (Fig. 7E)^71^.

## Discussion and Conclusion

In the realm of NPC structure and the mechanisms governing nucleocytoplasmic transport, a persistent and perplexing question remains: How do these FG Nups, composed of hundreds of copies and thousands of disordered protein segments rich in FG repeats, collectively construct a selectively permeable barrier that facilitates the crucial trafficking between the cytoplasm and nucleus? However, acquiring a comprehensive grasp of the FG-Nup barrier presents a compelling challenge due to the inherently dynamic and disordered characteristics of the involved proteins. In this manuscript, we harness the formidable capabilities of super-resolution SPEED microscopy and HaloTag labeling techniques, enabling us to delve into the intricacies of NPCs within living cells. Our study is centered on the exploration of the spatial distribution and dynamic behaviors exhibited by individual mammalian FG-Nups within living cells, offering valuable insights into their 3D arrangement and subsequently their functions in the nucleocytoplasimc tranport mechanism.

### Distinct dynamics, configurations, and arrangements of FG-Nups

In live cell observations, we have uncovered a remarkable diversity in the dynamics, configurations, and arrangements of FG-Nups within the NPC. Specifically, the dynamic extension, orientation, and spatial distribution of the FG-domains within the eleven FG Nups are notably influenced by their anchoring positions within the NPC. What is particularly intriguing is the identification of two distinct classes of extension patterns among FG-Nups. FG-Nups with an asymmetric positioning, either on the cytoplasmic or nuclear basket sides of the pore, display relatively less or unrestrained behavior. Conversely, centrally anchored symmetric FG-Nups, with the exception of Nup98, tend to have more limited extensions. Additionally, we have observed that different FG-domains align themselves along two primary conformation axes: either projecting outward from the NPC toward the cytoplasm/nucleus or orienting themselves toward the central axis of the NPC.

In the case of asymmetric FG-Nups positioned on the cytoplasmic and nuclear faces of the pore, the FG-domains primarily extend outward from the NPC into the cytoplasm or nucleus, occasionally orienting towards the NPC axis. This observation aligns with earlier EM studies, which proposed that the FG-termini of cytoplasmic- and nuclear-side FG Nups could project into their respective cellular compartments^31,97,98^. Specifically, this agreement between the EM study and our measurements is evident in the 40-nm central scaffold region. However, our findings also diverge from those obtained through EM studies. We observed that these FG Nups extend significantly deeper into the adjacent compartments in live cells, with an average extension length of approximately 80–130 nm into the cytoplasm and about 60–100 nm into the nucleus. When combined with the 40-nm central scaffold, this totals a maximum length of approximately 270 nm. These extension ranges are notably larger than the previously reported results, where eight cytoplasmic filaments were suggested to extend approximately 30–90 nm into the cytoplasm, and a basket structure was estimated to extend around 50–75 nm into the nucleoplasm, resulting in a total length of approximately 200 nm^1,32,37,43–46^. These differences could partially stem from the nature of the samples being investigated, as our study focuses on live cells, while EM studies often probe dynamics in fixed or frozen samples.

Of particular note is the intriguing “plug”-like appearance of Nup214 at an approximate midpoint of the cytoplasmic extension, spanning between 60 nm and 80 nm. Nup214 aligns itself with hCG1 and Nup385, forming a cap-like structure that covers the cytoplasmic entrance. Similarly, Nup153 contributes its domains to create a cap-like structure, in collaboration with Nup50 and TPR, positioned at the end of the nuclear basket within the range of 90–125 nm. This structural arrangement closely resembles the nuclear distal structure observed through EM^99^. Furthermore, the cap-like structure observed on both ends of the NPCs in mammalian cells, as depicted in this mapping, corresponds to the lobe-like structure previously observed in yeast NPCs^36^.

On the other hand, centrally located symmetric FG-Nups, including Nup54, Nup58, Nup62, and Nup98, predominantly occupy the central scaffold region of the NPC. They extend towards the central axis of the NPC, forming a structure reminiscent of a doughnut. This arrangement consists of a densely packed periphery abundant in FG domains, while the central region exhibits a lower spatial density of FG domains. Notably, Nup98, in addition to contributing significantly to the formation of the doughnut-shaped structure within the central scaffold region, also displays noteworthy overlaps with all other FG/FxFG Nups. This observation suggests that this particular GLFG-rich Nup not only plays a central role in shaping the doughnut-like structure but may also collaborate with other FG/FxFG Nups throughout the entire nucleocytoplasmic transport process, spanning from one end of the NPC to the other.

### Different roles of FG Nups played in mediating the nucleocytoplasmic transport

Considering the distinct configurations and spatial arrangements of FG Nups obtained from native NPCs, we have correlated them with the specific transport routes utilized by various molecules during their passage through the NPC. This correlation yields valuable insights into the roles that these FG Nups play in the nucleocytoplasmic transport process.

First, let us delve into the doughnut-like trichoid channel formed by the five central FG Nups at the central scaffold of the NPC. This channel boasts the highest spatial density of FG repeats and represents a more stable structure within the NPC. Almost all tested NTRs, including Imp β1, Imp β2, NTF2, CRM1, TAP-p15, and CAS, can efficiently interact within this channel^4,55,56,84–86^. Remarkably, these NTRs predominantly traverse the periphery of the channel, with limited occupancy at its center. This inclination towards the periphery is primarily driven by hydrophobic interactions with the peripheral FG domains. Meanwhile, the central region with a diameter of about 7 nm within the doughnut-shaped channel, which contains fewer FG domains and is less occupied by NTRs, serves as the primary pathway for the passive diffusion of small molecules while concurrently acting as a barrier to hinder the diffusion of signal-absent large molecules.

In contrast, the configurations formed by the asymmetric FG Nups located at both ends of the NPC exhibit significant conformational variability. These FG Nups possess considerable flexibility, enabling them to extend deeply into both the cytoplasmic and nuclear compartments. This adaptability proves advantageous for different NTR-assisted transport scenarios. For instance, owing to their flexible lengths, these FG Nups can offer binding sites deep within neighboring compartments for NTRs, facilitating effective NTR docking onto the NPC. The inherent flexibility of their disordered domains also allows for the maximum exposure of hydrophobic interaction sites for NTR binding. Observations in live and permeabilized cells have revealed distinct pathways for various importins and exportins through the NPCs, particularly differing in their routes at both ends of the NPC^10,56^. Previous *in vitro* analyses have outlined how specific NTRs can effectively interact with particular FG Nups while also sharing some FG Nups with other NTRs to navigate the NPC^4,5,100–102^. Consequently, we have previously demonstrated that competitions indeed occur among these NTRs in native NPCs, with Imp β1 for importing and CRM1 for exporting outcompeting other NTRs^56^.

Remarkably, the cap-like structures formed by these asymmetric FG Nups at both ends of the NPC, which can adopt open and closed states, serve not only as interaction sites for both importins and exportins (functioning as docking or releasing sites) but also act as a primary barrier, preventing large particles from passively diffusing into the NPC (Supplementary Fig. S12-S13). Interestingly, both the cap-like and the doughnut-like structures within the NPC exhibit dual functionality, providing major interaction sites for NTRs while simultaneously facilitating or impeding passive diffusion.

### Revisiting existing nuclear transport models based on the 3D organization of FG Nups and distinct transport routes

The presence of intrinsically disordered regions within FG-Nups poses a challenge in comprehending their structural arrangement and functional roles in nucleocytoplasmic transport. Over time, various strategies have been employed to investigate the complex organization and behavior of FG-Nups within the NPC, resulting in the development of multiple nuclear transport models that contribute insights into the structural layout and functional importance of FG-Nups. Each model holds merits within specific domains. However, it is essential to recognize the absence of definitive confirmation for any of these models, as supported by empirical evidence. Furthermore, these models often give rise to conflicting viewpoints and differing proposals.

Our objective here is not to assess the validity of any particular model but rather to harmonize these new findings with the attributes delineated by each existing model, with the anticipation that this alignment can advance our comprehension of nucleocytoplasmic transport mechanisms. As illustrated in Supplementary Table S8, each of these existing models has emphasized unique structural features and mechanisms, resulting in a spectrum of proposed interpretations. To the best of our comprehension of these existing models, we have exclusively documented the suggested transport pathways for passive diffusion and facilitated transport, along with the forecasted FG Nups that contribute to the NPC’s selectivity. Specifically, these predictions were classified into three categories: 1) Is there a single or multiple channel(s) at the NPC’s center for passive diffusion? 2) Are there distinct passive and facilitated pathways through the NPC? 3) Do FG Nups at the NPC’s center and/or both ends play a role in the NPC’s selectivity? And 4) Is there a “plug” within the center of NPC?

We have included these predictions because we are confident that our employed methodologies effectively distinguish between these proposals. Over the course of more than a decade, our lab has utilized high-speed single-molecule localization microscopy and virtual 3D super-resolution techniques to map passive diffusion and facilitated transport routes, as well as the native configurations of FG Nups^10,11,55,56,62,71,103^. Based on our findings, we have verified the presence of a singular channel located at the center of the NPC, herein referred to as the “doughnut-like channel” in this manuscript. The central region of this channel functions as the primary diffusion pathway for small molecules, while its FG-rich periphery is utilized during NTR-facilitated transport^47,50–52^. Furthermore, our observations consistently reveal that passive diffusion and facilitated transport adopt separate routes through both the NPC’s center and either end. Models that align with these observations encompass the “Oily Spaghetti” and “ROD” models.

Regarding the NPC’s selectivity, we propose that FG Nups located at the central, cytoplasmic, and nuclear sides collectively contribute to its selective function. Specifically, both the cap-like and doughnut-like structures within the NPC serve dual roles, serving as significant interaction sites for NTRs while simultaneously facilitating or impeding passive diffusion. Our observations reveal that small molecules can easily diffuse through the NPC, whereas larger molecules encounter obstacles at both the cap-like and doughnut-like structures of the NPCs. In contrast, NTR-facilitated transport enables large cargo molecules to effectively overcome these barriers and transit through the NPCs. These findings align with the theoretical frameworks proposed by previous models, such as the “Virtual Gating/Polymer Brush” models. These models postulate the existence of an entropic barrier formed by the polymer-brush-like of FG-Nups, characterized by weak FG–FG interactions at both ends of the NPC, which impede the passive diffusion of large molecules. Conversely, large NTR–cargo complexes surmount the entropic penalty associated with the excluded volume effect at the NPC’s scaffold by gaining enthalpy from NTR–FG interactions.

Furthermore, we want to emphasize that we do not rule out the possibility that the passive diffusion of small molecules and the effective interactions between NTR-cargo complexes and FG Nups, especially within the cap-like structures of the NPC, can be alternatively explained by the theoretical framework proposed in the “Selective Phase/Hydrogel” models. According to these models, FG-Nups form a meshwork, with hydrophobic FG repeats interacting among themselves, resulting in small gaps within this meshwork. These gaps permit the passive diffusion of small particles, while larger NTR-cargo particles must interact with the hydrophobic regions through NTR-FG interactions to displace FG-FG interactions, thereby facilitating their transport through the pore^104,105^. However, it is crucial to underscore that the presence of the “doughnut”-like structure within the NPC’s central region suggests that the pathways for passive diffusion and facilitated transport occupy distinct spatial domains, with significantly less overlap than suggested by the “Selective Phase/Hydrogel” models.

Finally, both earlier and recent studies have raised the possibility of a central plug, often referred to as a central transporter, within the NPC^89,98,106,107^. This structure has remained enigmatic and contentious, sparking substantial debate regarding its composition and function^3,32,47,57,78^. While the precise nature of the plug has remained elusive, a consensus has emerged that it represents a dynamic entity that manifests itself only under specific conditions^32,108–110^. Certain hypotheses posit that these plug-like formations could arise from the inadvertent sequestration of substantial cargo during imaging procedures, as proposed by Stoffler et al.^106^. However, a recent study conducted by Li et al.^111^, utilizing cryo-EM to examine pre-60S particles ensnared within yeast NPCs, has introduced captivating insights. These significant particles seem to exhibit a preference for tracing the peripheral contours of the pore structure, departing from a central trajectory. This observation casts doubt on the notion that large transiting cargos are the definitive source of the observed plug phenomenon.

The cap-like structures, which we have observed at both extremities of the NPC in live cells, offer further potential insights into this model. Techniques such as EM and cryo-EM typically capture fleeting snapshots of the dynamic nuclear transport processes within NPCs. The existence of cap-like structures in a closed state might be construed as indicative of a ‘plug’ within the NPC, whereas imaging these structures in an open state could yield an alternate interpretation. Analogous situations may occur when dealing with large cargo complexes within NPCs, where they may either be captured or elude detection during measurements. Consequently, we contend that none of the prior observations necessarily stand as inaccuracies; instead, they represent distinct facets of reality, each emerging under specific conditions and within the confines of particular methodologies. To gain a comprehensive understanding of these phenomena, it becomes imperative to amalgamate the strengths of multiple methods and techniques.

### Distinct Pathways through the Capped Adaptable Trichoid Channels (CATCH) within the NPC

To this end, our measurements revealed the dynamic confirguration of FG Nups and how they form a selectivity permeable barrier to mediate passive and facilitated diffusion through the NPC. All the major features can be summarized in such a schematic that distinct pathways through the capped adaptable trichoid channels (CATCH) within the NPC.

In the context of the CATCH model, during NTR-assisted nuclear import, NTRs initially interact with the cap-like structure formed by FG domains on the cytoplasmic side of the NPC. These interactions can occur at a distance of up to 150 nm from the NPC’s right center. The concentration of NTRs at the entrance plays a crucial role in facilitating their entry into the NPC, as a higher concentration helps them outcompete other NTRs by occupying more binding sites. Additionally, the conformational changes of the cap-like structure may be influenced by the local NTR concentration as well. Once NTRs reach the central doughnut-like trichoid channel, guided by the high concentration of FG domains at the channel periphery, they smoothly move through the periphery rather than locating at the channel’s center. Some NTRs may need to interact with the cap-like structure formed in the nuclear basket before they can facilitate the disscoation of cargo into the nucleus from the NPC, while others may bypass this interaction and directly enter the nucleus.

A similar scenario can occur for NTR-assisted nuclear export, where some NTRs first dock their cargos at the cap-shaped structure in the nuclear basket. Once again, a cooperative action involving more NTRs enhances the movement of cargos, particularly large ones, into the central channel effectively. Similarly, the exporting complexes primarily utilize the periphery of the central channel. Interestingly, recent cryo-EM data has confirmed that even for almost the largest exporting complexes, such as pre-ribosomal subunits, the choice of the peripheral path holds true^111^. It appears that most exporting NTRs will interact with the cap structure on the cytoplasmic side of the NPC before they are released into the cytoplasm.

In the CATCH model, the scenario for passive diffusion is straightforward. Specifically, small molecules (< 40–60 kDa) either pass through or navigate around the cap-like structure and continue to diffuse through the less FG-occupied central axial channel. Conversely, larger particles (> 60 kDa) initially encounter an obstacle within the cap-like structure and subsequently face hindrance within the narrower central channel with a diameter of approximately 7 nm.

### Advantages and limitations of the approach

Our virtual 3D super-resolution imaging technique, SPEED microscopy, offers several distinct advantages compared to other 3D super-resolution microscopy methods. Firstly, SPEED microscopy enables the simultaneous capture of fast dynamics and sub-micrometer subcellular structural details in three dimensions, all achieved with high spatial and temporal resolution (Supplementary Table S2). Secondly, SPEED microscopy simplifies sample preparation compared to other single-molecule localization microscopy techniques, as it does not necessitate specialized fluorophores. Moreover, SPEED microscopy finds extensive utility in live cell imaging due to its pinpointed on-off illumination pattern, which minimizes phototoxic effects and reduces out-of-focus background fluorescence. Collectively, these characteristics position SPEED microscopy as an ideal choice for live-cell imaging applications.

However, it is important to note some operational and application limitations of this method. Firstly, achieving the alignment of an inclined illumination requires expertise in optics or microscopy. In SPEED microscopy setup, we present an alternative vertical illumination pattern that reduces the technical demands and is simpler to implement. Additionally, the current functional range of our 2D-to-3D transformation algorithm is confined to the retrieval of 3D dynamics and structural data from rotationally symmetric systems. Examples include primary cilia^112^, NPCs^55,56,62^, and glass nanocapillaries (GNCs)^113^. This constraint arises because our 2D-to-3D transformation algorithm assumes that the distribution in the θ dimension remains constant at each (x, r) in rotationally symmetric systems (as depicted in Supplementary Fig. S9-S11). Furthermore, this assumption leads to an averaging of finer details within each radial bin along the r dimension, typically at the scale of 5–10 nm. Lastly, successful implementation of this method necessitates expertise in coding and modeling to ensure the reproducibility of the acquired 3D structural data.

## STAR Methods

### Cell line, cell culture, and transfection

A stable HeLa cell line expressing the mCherry conjugate of POM121 were utilized in this study (RRID: CVCL_A916). Cells were cultured in DMEM medium containing 10% FBS and 1% penicillin-streptomycin at 37°C in an incubator with 5% CO_2_. For live cell microscopy experiments, cells were transfected using TransIT^®^-LT1 reagent (Mirus Bio LLC, Madison, WI) with plasmids expressing EGFP and HaloTag-tagged Nups. Transfected cells were cultured in DMEM medium for approximately 24 hours (except for Nup62, Nup58, and Nup54, which required around 48 hours) before being incubated with transport buffer (20 mM HEPES, 110 mM KOAc, 5 mM NaOAc, 2 mM MgOAc, and 1 mM EGTA, pH 7.3) prior to imaging. The transfection efficiency via transient transfection was assessed for Halo- and EGFP-tagged FG Nups, revealing that approximately 80% of the native FG Nups were replaced with the labeled Nup per pore (refer to Fig. S2). Subsequently, the cells were reintroduced into growth media and monitored for two rounds of division after imaging to assess cell viability.

In the case of imaging in permeabilized cells, cells were grown and transfected following the same procedure as described for live cell imaging. Subsequently, cells were treated with transport buffer containing 40 μg/mL digitonin for 3 minutes and washed with transport buffer containing 1.5% polyvinylpyrrolidone (PVP; 360 kDa) at least three times to prevent osmotic swelling of the nuclei immediately before imaging. More details can be found in Supplementary Information. For living-cell tracking of NTRs through the NPCs, HeLa cells were transfected with EGFP-tagged Imp β1, Crm1 or TAP on their C termini.

### Plasmid design and construction

The original plasmids containing Nup50 (P30482), Nup58 (P30483), Nup62 (P30484), Nup98 (P30487), Nup153 (P30457), Nup214 (P30488), hCG1 (P30476), and POM121 (P30554) were obtained from the European Saccharomyces Cerevisiae Archive for Functional Analysis (Euroscarf, Institute for Molecular Biosciences, Johann Wolfgang Goethe-University Frankfurt, Germany). The pEGFP-Tpr102 plasmid was purchased from Addgene (Cambridge, MA), while pEGFP-FL-Nup358^112,113^ was generously provided by Dr. Jomon Joseph (National Centre for Cell Science, Pune University, India). The Nup54 sequence (GenBank NM_017426 nucleotides 142–1665) was synthesized by Genewiz Inc. (South Plainfield, NJ). The pT7-EGFP-C1-HsNXF-1_K (eGFP-TAP) plasmid was acquired from Addgene (Cambridge, MA) and was deposited there courtesy of Dr. Elisa Izaurralde’s lab^114^.

The DNA sequences of the Nup proteins (except for Nup358 and TPR) were amplified from the original plasmids and linked with EGFP sequences either at the C-terminus or N-terminus. The EGFP-Nup sequences were inserted into the multiple cloning sites (MCS) of pHTC HaloTag^®^ CMV-neo vectors (for C-terminal HaloTag expression, Promega Corporation, Madison, WI), and Nup-EGFP sequences were inserted into the MCS of pHTN HaloTag^®^ CMV-neo vectors (for N-terminal HaloTag expression). This resulted in the generation of plasmids that express Nup proteins with EGFP and HaloTag on opposite sides.

For plasmids expressing tagged Nup358, the HaloTag sequence was amplified from the pHTC HaloTag^®^ CMV-neo vector and inserted into the C-terminal region of Nup358 on the original pEGFP-FL-Nup358 plasmid, creating the pEGFP-Nup358-HaloTag plasmid. Additionally, we inserted the HaloTag sequence before the EGFP sequence on the original pEGFP-FL-Nup358 to generate the pHaloTag-EGFP-Nup358 plasmid. The Gibson assembly method was employed to generate pEGFP-Tpr-HaloTag and pHaloTag-Tpr-EGFP plasmids.

### Janelia Fluor^®^ 646 HaloTag^®^ ligands (JF646)

Janelia Fluor^®^ HaloTag^®^ ligands represent state-of-the-art chemical probes meticulously crafted for precise labeling of HaloTag fusion proteins in living cells^60^. Leveraging their cell-permeable nature, these ligands effortlessly enter live cells, enabling real-time visualization of protein dynamics and interactions. Renowned for their exceptional brightness, photostability, and narrow emission spectra, Janelia Fluor^®^ dyes shine in single-molecule tracking and super-resolution imaging. In our experiments, live cells were labeled by adding JF646 to the growth medium and incubating the samples for 15 minutes. Labeling concentrations typically ranged from 100 to 500 nM for confocal microscopy and from 5 to 50 nM for single-molecule tracking experiments.

### Laser scanning confocal microscopy

FV3000RS laser scanning confocal microscope (FV3000RS; Olympus, Shinjuku, Tokyo, Japan) was used for all our confocal microscopy experiments. The FV3000RS confocal microscope consists of Olympus IX83P2ZF equipped with a 1.4-NA 60X oil-immersion low chromatic aberration objective (PLAPON60XOSC2; Olympus, Shinjuku, Tokyo, Japan), 20-mW 488-nm continuous wave solid-state lasers (Coherent OBIS), Galvanometer Scanner with the scanning resolution from 64 × 64 to 4096 × 4096 pixels and scanning speed from 2 μs - 1000 μs per pixels, single motorized pinhole with pinhole diameter of ø50–800 μm. a Cooled GaAsP photomultiplier, and the FV3000 system software (FV31S-SW; Olympus, Shinjuku, Tokyo, Japan) for data acquisition and processing. The EGFP fluorophore was excited by 488-nm laser, mCherry was excited by 561-nm laser, and the JF646 was excited by 633-nm laser. Live cells were scanned at the scanning resolution of 2048 × 2048 (104 nm/pixel) and 4096 × 4096 pixels (52 nm/pixels) respectively, and at scanning speed of 2 μs per speed. Cells were imaged while living and temperature was controlled to be 37°C.

### High-speed single-molecule SPEED microscopy

High-speed single-molecule microscopy was performed with an Olympus IX81 equipped with a 1.4-NA 100× oil-immersion apochromatic objective (UPLSAPO 100×; Olympus, Shinjuku, Tokyo, Japan), a 50-mW 488-nm semiconductor laser (Coherent OBIS, Santa Clara, CA), a 50-mW 561-nm laser (Coherent OBIS), an on-chip multiplication gain CCD camera (Cascade 128+; Photometrics, Tucson, AZ), and the Slidebook software package (Intelligent Imaging Innovations, Denver, CO) for data acquisition and processing. An optical chopper (Newport) was used to generate an on–off mode of 561-nm laser excitation. EGFP, mCherry, and JF646 were excited by 488, 561, and 633 nm lasers, respectively. Single molecules were detected at 2 ms and 50 ms per frame. The chromatic aberration between three colors (blue, green and red) channels was experimentally determined to be ~2–3 nm by measuring hundreds of Alexa Fluor-dye-labeled EGFP fluorescent molecules immobilized on the surface of the coverslip. A diffraction-limited single-point illumination is focused to the focal plane during the experiment, and the illumination intensities at the focal plane were approximately 100–150 kW/cm^2^ (488 nm, 561 nm, and 633 nm).

SPEED microscopy fills the technique niche of capturing single molecules transport through sub-diffraction-limit bio-channels at high spatial (<10 nm) and temporal (0.4–2 ms) resolution. While this technique is capable of exceptional temporal resolution, this manuscript primarily made use of 50 ms temporal resolution. This temporal resolution was utilized for three reasons. First, it permitted unbound, fast moving, dyes to pass through the detection area without being recorded. Second, the long collection time permitted the collection of sufficient photons to permit an exceptional spatial resolution less than 10 nm. Third, this time interval provided an important internal control confirming that only single-Nups anchored to the NPC were evaluated.

The exceptional temporal and spaial resolution of SPEED microscopy is achieved through four main technical modifications on traditional epifluorescence, or confocal light microscopy. (1) A small vertical illumination point spread function (PSF) is used for the excitation of single molecules within the focal plane. The small illumination greatly increases the allowable detection speed by reducing the number of camera pixels required for detection. Also, this small illumination helps to avoid out-of-focus fluorescence greatly reducing the background noise to increase the signal to noise ratio. (2) A high optical density can be achieved (100–500 kW/cm2) via this small illumination volume which causes a high number of photons from the fluorophores to be emitted (3) A collection of a high-number of photons in a short time (2–50 ms) period increases spatial resolution by reducing the effects of diffusion on the single-molecule localization precision of moving molecules (4) Using a pinpointed illumination in live cells will also cause less photo-toxicity compared to wide-field setups. Frame rates of 50 ms were employed to capture single FG-Nups anchored to the NPC. For freely diffusing JF dye or transiting Nups through the NPC, 2ms was utilized. The 2D gaussian fitting of single-molecules was conducted using either GDSC SMLM ^114^, or GLIMPSE software ^115^. Data was then filtered using time and precision to avoid unnecessary shifts in the data and unwanted high precision points that may increase the noise in the final data. The precision cutoff was ~10nm for all experiments. A detailed protocol can be found in Nature Protocols^61^.

### Alignments between different imaging approaches on the same light microscope.

In some of our experiments, cell samples were subsequently imaged by wide-field transmission light and inverted beam-expanded or -focused lasers. Any misalignments between these different imaging modes were determined by comparing images of the same sample taken by these methods and later corrected in data analyses. In detail, first, we brought the equator of nuclear envelope (NE) to the focal plane under wide-field transmission light mode and took an image of NE. Then, we switched to wild-field, narrow-field or single-point fluorescence mode to image the same NE. Third, we localized the NE in both images and calculated any shifting errors. The errors were corrected in later data analyses by overlaying the NE captured in each type of light microscopy, thereby eliminating any shift.

### Localization of the nuclear envelope (NE)

The position of the NE was determined with subdiffraction limit accuracy by fitting the fluorescent or bright-field images of the NE. Gaussian fits were applied to pixel intensities within rows or columns approximately perpendicular to the NE. The peak position of the Gaussian corresponding to a specific set of pixel intensities denoted the NE position for that particular row or column. These peak positions, obtained from a series of such Gaussians, were further fitted with a second-degree polynomial. This polynomial fit provided the orientation of the NE across the entire image. The localization precision of the NE’s middle plane was determined to be approximately 10 nm.

### Localization precision of fluorescent spots.

The localization precision for fluorescent NPCs, as well as moving fluorescent macromolecules, was defined as how precisely the central point of each detected fluorescent diffraction-limited spot was determined. Fluorescent NPCs were fitted with a 2D elliptical Gaussian, and the localization precision was determined by the standard deviation (s.d.) of multiple measurements of the central point. For moving molecules, the influence of particle motion during image acquisition should be considered in the determination of localization precision. In detail, the localization precision for moving substrates (σ) was determined by the equation

$$\sigma =\sqrt{F[\frac{16\left(s^{2}\;+\;a^{2}/12\right)}{9N}+\frac{8\pi b^{2}{\left(s^{2}\;+\;a^{2}/12\right)}^{2}}{a^{2}N^{2}}]}$$

where F is equal to 2, N is the number of collected photons, a is the effective pixel size of the detector, and b is the SD of the background in photons per pixel, and $s=\sqrt{s_{0}^{2}+\frac{1}{3}D\Delta t}$, where s0 is the SD of the point spread function in the focal plane, D is the diffusion coefficient of substrate, and Δt is the image acquisition time. Calculation of the diffusion coefficient was performed by plotting on a mean-square displacement versus time. The data was fitted with $MSD=4D\Delta t^{\alpha }$.

The precision, determined from the standard deviation of multiple measurements and the provided equation, was validated by examining the localizations of immobile fluorescent molecules and fluorescent NPCs. Specifically, 230 immobile Alexa Fluor 647-labeled EGFP molecules were measured, and the two methods yielded a difference of 0.5 ± 0.1 nm. In our measurements, typically millions of photons from the 8 copies of mCherry- or EGFP-fused to 8 copies of POM121in an NPC were collected by using an optimized laser excitation power within 2–3 seconds. The collected millions of photons enabled us to achieve a spatial resolution of approximately 1–3nm Moreover, any potential drift in the NPC or the stage during the 3-second detection window was minimal, measuring less than 1 nm and thus ensuring the accuracy of our measurements. The introduced localization error, resulting from overlaying 2D single-molecule trajectories of FG-Nups onto the POM121 centroid, followed the relationship $\delta_{combined}=\sqrt{{\delta^{2}}_{FG-Nup}+{\delta^{2}}_{POM121}}$, where δ represents the localization precision.

### Determinations of NPC’s centroid

Before capturing single-molecule videos, a single mCherry- or GFP-labeled NPC was imaged to serve as a reference point for the cells. To ensure the accuracy of the NPC’s centroid representation, both the initial integrated fluorescence intensity before photobleaching and the subsequent photobleaching steps for POM121 were recorded. These measurements can confirm that the stably expressing HeLa POM121-mCherry cell line (RRID: CVCL_A916) indeed contained 8 labeled copies of POM121, as illustrated in Fig. S1. A detailed protocol outlining the counting of photobleaching steps can be found in Current Protocols^116^. We typically completed the imaging of labeled NPC or NE within 2–3 seconds and the subsequent recording of single-molecule video within 120 seconds in a live cell. After single-molecule recordings, the image of the single NPC or the NE were collected again. Upon comparing images captured before and after single-molecule recordings, we noted minor drifts of approximately 5–6 nm in both the specimen stage and the objective during the detection period. These drifts were corrected in subsequent data analyses. However, single-molecule recordings were discarded if the drifting error exceeded 10 nm. Utilizing the NPC image, the NPC’s centroid was fitted using GDSC. Subsequently, the single-molecule traces collected for discrete Nups were zero-normalized to the determined centroid, allowing for the overlap of multiple NPCs.

### Superimposing 2D single-molecule spatial data over the NPC centroid

The centroid of the NPC, as described in the section detailing localization precision, is employed to overlay the spatial locations of the FG-Nups obtained. Following these superimpositions, the data was further validated using X and Y histograms to account for drifting and other potential sources of error. A flowchart illustrating this process can be found in Supplementary Fig. S17.

To begin, data for each cell was corrected in the Y dimension. This correction involved fitting the data with either a single or double symmetrical Gaussian function. Subsequently, the data was adjusted to center the peak position at zero on the Y-axis of the Cartesian coordinate system. The Y dimension data, ideally symmetrical due to the cylindrical NPC geometry, was thus aligned. Next, in the X dimension, the spatial gap in data points caused by the nuclear envelope was centered to the zero position. This alignment was achieved through Gaussian fitting of the data. After collecting a sufficient amount of data from multiple cells, ensuring it was about 90% reproducible through the 3D reproducibility algorithm, histograms were generated. These histograms were instrumental in centering the data in both X and Y dimensions. Errors from Gaussian peak fitting were obtained for the final distributions. The ultimate error in centering the data was calculated by adding the final fitting errors obtained for both the X and Y dimensions. This detailed process is illustrated in Supplementary Fig. S18.

### Obtaining extension length from diffusion profiles

Mean square displacement (MSD) plots were obtained similar to previously published methods ^116^. Trackmate software ^117^ was utilized through FIJI ImageJ ^118^ to obtain single-molecule trajectories. From a total of 50 trajectories obtained for each terminal end of each FG-Nup, mean square displacement plots were generated. Mean square displacement calculations were made using the MSD analyzer package for MATLAB ^116^. Individual trajectories for each Nup terminus were summarized into a single MSD curve using the weighted mean based on trajectory length, and the summarized MSD curve was fitted to the power function $MSD(t)=4{Dt}^{\alpha }$; where t is the time elapsed, MSD⁡(t) is the mean square displacement as a function of time, D is the diffusion coefficient, and α is the diffusive exponent. Due to the nature of the power function $MSD(t)=4{Dt}^{\alpha }$, log-log fitting (log⁡(MSD(t))=log⁡(4D)+αlog⁡(t)) was performed, as α is equal to the slope of the linear result of log-log fitting, while diffusion coefficient could be calculated by 10^intercept^/4. Analysis used the published threshold for strong subdiffusion of ≤ 0.3^66^.

Tracks with confined displacement (those with a diffusive exponent < 1) were used to obtain the particle’s average extension and max extension length. Average extension length using the square root of the MSD value at which the power function plateaus (typically when t = 1s). In contrast, the max extension length recorded the most extended possible movement length for each trajectory. Both extension lengths were plotted as histograms, as shown in Figure 2 and Figure S6-S7, and fit using a Gaussian distribution with the peak values recorded in Table 1.

### Simulation of confined diffusion

A monte carlo simulation where an open ended cylinder analog of the NPC central channel with a length of 40 nm and a 25 nm radius. A ball and stick analog of a Nup was generated where FG regions were balls and the IDR between each FG region regulated the maximum distance that each “ball” could difuse away from one another. The simulation was generated with 8 copies of the Nup analog having root positions randomly determined along the axis of the NPC cylinder. Prior to capturing localizations of the “fluorophore” at the terminus of the Nup analog, the first 10 seconds of random diffusion were discarded to prevent positional bias. After discarding the initial positioning, the simulation ran at the determined rate (0.5 ms, 1 ms, 5 ms, 10 ms, 20 ms, 30 ms, 40 ms, 50 ms, 100 ms, 200 ms, 500 ms, and 1000 ms) for 10 seconds. The simulation code can be found at: https://github.com/YangLab-Temple/Master/tree/2023-Single-Nup-Manuscript/2023%20Single%20Nup%20Manuscript/Nup%20Analog%20Simulation

Once the localization baseline at a capture rate of 0.5 ms was generated, the locolization at 50 ms capture rates were mathematically calculated by averages every 100 localizations to mimic the signals received by the camera. The simulated localizations at 50 ms capture rate were used to calculate the MSD and diffusion coefficient using the same MATLAB addon mentioned in the previous section. The simulation parameters were adjusted accordingly until the diffusion coefficient of the simulated particle was slightly faster (~10%) than the fastest-moving Nup termini observed in our experiment (shown in Fig 2, Supplementary Fig S5 A). This ensures that our simulation is fast enough to represent the Nup termini we observed in this project.

With adjusted simulation parameters, a new set of localizations were generated at 0.5 ms capture rate, and the localizations corresponding to different interval at 1 ms, 5 ms, 10 ms, 20 ms, 30 ms, 40 ms, 50 ms, 100 ms, 200 ms, 500 ms, and 1000 ms, were calculated. The 2D to 3D transformation algorithm was then applied to each interval group, and the R peak position was obtained using Gaussian fitting, the same as the method described in the 2D to 3D transformation section.

### Algorithms for 2D to 3D transformation to generate 3D probability density maps of FG Nups

The detailed transformation process used to compute the 3D spatial probability density maps of particles transiting through the NPC was described in our previous publications ^56,61,62,119,120^ and demonstrated again here in Supplementary Fig. S9 - S11 and Fig. S14 – S20. In short, the 3D spatial locations of molecules transiting through the NPC can be considered in either Cartesian (X, Y, Z) or cylindrical (X, R, Ɵ) coordinates. In microscopic imaging, the observed 2D spatial distribution of particle localizations is a projection of its actual 3D spatial locations onto the XY plane. The underlying 3D spatial distributions can be recovered by projection of the measured Cartesian (X, Y) coordinates back onto the simplified cylindrical (X, R, constant) coordinates, based on the expected cylindrically symmetrical distribution along the Ɵ direction of the nuclear pore. XY data is grouped into X-dimensional bins each containing at least 200 locolizations. This number was determined based on our lab’s earier works on reproducibility (as shown in Supplemtnal Fig. S15)^70^, that 200 locolizations could maximixing the chance of getting 90% higher reproducibility wihout sacrifacing a lot on X axial resolution. These XY data groupings are then utilized to calculate probability density maps representing the X and R dimensions the NPC. This is possible because for a radially symmetrical cylindrical geometry it does not matter whether imaging was performed from the XY (side view) or the YZ (radial cross section) plane. The obtained Y dimensional histograms are ideally identical (See Supplementary Fig. S10A). Thus from the data points collected along the axis of the NPC, the densities in the radial dimension can be obtained by solving the matrix equations provided in Fig. S6 and references^61,120,121^. The R dimension bin sizes were determined either through Chi-square error and P-value analysis (Supplementary Fig. S15) or based on the consistency of the R dimension peak position at various R bin sizes. The resulting 3D, surface-rendered visualizations shown in the figures were generated with code written in Python 3.9^122^ with various packages^123–125^.

The actual reproducibility of each X-dimensional bin was then calculated using Monte Carlo simulations. Simulations were conducted using randomly sampled X and Y positions confined to cylinders of varying radii corresponding to those obtained experimentally listed in Supplementary Table S2. Each sampled point was calculated using a probability distribution corresponding to our experimental single molecule localization precision to simulate error. The data was then run through our X, Y to X, R transformation process. Ten thousand iterations were run under the necessary conditions for each experiment to determine the minimum amount of data needed to resolve a 3D spatial transport route with an acceptable reproducibility percentage. For the reproducibility percentage, the proportion of simulated data sets that fell within the acceptable range was calculated (See Supplementary Fig. S14 and Table S2). The script to perform the 2D to 3D transformation is provided at https://github.com/YangLab-Temple/Master/tree/master/Python%202D-to-3D/Python. A detailed protocol to perform this transformation is found at Nature Protocols ^61^.

### Localization precision for 3D probability density maps of FG Nups and transport routes of various transiting molecules

To ensure a high reproducibility of 3D spatial probability density maps obtained for each candidate, extensive measurements were conducted by combining experimental data and computational simulation. It is important to note that route localization precisions are different from single-molecule localization precision. In detail, the route localization precision is determined by two parameters: one is the number of single-molecule locations and the other is single-molecule localization precision. As shown in Supplementary Figure S14, simulated data was used to estimate the minimum number of single-molecule localizations required to generate a reliable 3D probability density map for routes of 25 nm (central channel transport) or 40 nm (peripheral channel transport) radial distances. A single-molecule localization precision of 10 nm was used to reflect the precision of our experimentally collected data. We used three different sample sizes (100, 200, and 500 points) and converted the 2D data to 3D by using our transformation algorithm. Peak positions were fitted for data generated from each of the three sample sizes. 100 fittings were used to determine the peak position and the standard deviation is used for the route localization precision. Simulation code can be found at: https://github.com/YangLab-Temple/Master.

### Reproducibility for 3D probability density maps of FG Nups and transport routes of various transiting molecules

The reproducibility for 3D probability density maps of FG Nups and transport routes was validated through a Monte Carlo simulation previously developed by our lab group. This simulation utilizes the calculated localization precision of single-molecule localizations in conjunction with the quantity of experimentally derived single-molecule localizations to calculate the reproducibility^70^. This simulation is open source and can be found here: https://github.com/YangLab-Temple/Master/tree/master/reproducibility%20rate. The reproducibility was summarized in Supplementary Table S2.

### Statistical validation of the cap-like structures formed on both ends of the NPC.

During our measurements, we observed variations in the presence (closed) or absence (open) of the “plug”-like structure associated with Nu214 and Nup153 among the NPCs we scanned. To overcome the limitation of scanning only ten different NPCs from ten separate live cells for each of these two FG Nups, we conducted a robust statistical analysis to determine the percentages representing the engaged (closed) and disengaged (open) states. To achieve this, we randomly selected several hundred spatial locations, ensuring a reproducibility rate of ⩾90%, from the total spatial locations collected across all measured NPCs. These selected 2D locations were subsequently transformed into 3D probability density maps. This process was repeated 10,000 times, and the resulting maps were analyzed to quantify the percentages of closed and open states.

### Statistics

Experimental error is reported as mean ± standard error of the mean, or if Gaussian peak fitting analysis was utilized, fitting error was applied. Overlap in X dimension are reported in Pearson correlation coefficient and Spearman’s rank correlation coefficient. Overlap in the R dimension is only evaluated if overlap in the X dimension already is present. The overlap is reported in Pearson correlation coefficient and Spearman’s rank correlation coefficient.

### Figure and plot generation

Plots used in this article were generated in OriginPro (version: 2019b; OriginLab, Northampton, MA, USA) software and Python 3.9^122^ with the scipy, matplotlib, and numpy packagtes installed^123–126^. The NPC structure used to display the scaffold structure of the NPC present in Figures 2–6 was collected from RCSB PDB^127^ (RCSB.org) of PDB ID 7TBJ^128^ created with Mol*^129^.

## Figures and Tables

**Table 1.** Peak Positions in X (along the nucleocytoplasmic transport axis) for Each FG-Nup, Along with Average and Maximum Extension Lengths. ‘(FG)’ indicates the terminus with a higher concentration of FG motifs. Nups are sorted based on the peak positions determined for the FG-rich terminus, with the FG-Nup farthest into the cytoplasm (with the most negative value) listed first and the FG-Nup farthest into the nucleus listed last.

Nup	Tag position	Peak X (nm)	Average Extension Length (nm)	Max Extension Length (nm)
**Cytoplasmic FG-Nups**				
Nup358	N	−48 ± 1	40 ± 1	46 ± 1
	C (FG)	−73 ± 2	71 ± 5	99 ± 6
Nup214	N	−68 ± 1	35 ± 1	45 ± 1
	C (FG)	−129 ± 10 (17%) −99 ± 1 (8%) −58 ± 2 (70%) 24 ± 3 (5%)	58 ± 3	82 ± 1
hCG1	N (FG)	−93 ± 4 (85%), 71 ± 15 (15%)	61 ± 7	83 ± 4
	C (FG)	−83 ± 12 (46%), −47 ± 2 (54%)	61 ± 5	90 ± 7
**Central Pore FG-Nups**				
Nup98	N (FG)	−45± 4 (50%), 46 ± 4 (50%)	67 ± 3	94 ± 3
	C	−19 ± 1 (50%), 19 ± 1 (50%)	25 ± 1	29 ± 1
Nup62	N (FG)	−12 ± 1	37 ± 1	51 ± 1
	C	−13 ± 1	19 ± 1	39 ± 1
Nup54	N (FG)	−8 ± 1	36 ± 1	57 ± 1
	C	−15 ± 1	23 ± 1	39 ± 1
POM121	N	1 ± 1	21 ± 1	28 ± 1
	C (FG)	0 ± 1	37 ± 1	42 ± 1
Nup58	N (FG)	11 ± 1	20 ± 1	30 ± 1
	C (FG)	−1 ± 1	33 ± 3	49 ± 2
**Nuclear Basket FG-Nups**				
Nup50	N (FG)	39 ± 1	55 ± 3	80 ± 5
	C	43 ± 1	45 ± 1	57 ± 1
Nup153	N	32 ± 1	26 ± 1	37 ± 1
	C (FG)	52 ± 2	59 ± 1	82 ± 3
TPR	N	31 ± 1	36 ± 2	52 ± 1
	C (FG)	54 ± 1	75 ± 6	108 ± 7
